# On the development of the chondrocranium and the histological anatomy of the head in perinatal stages of marsupial mammals

**DOI:** 10.1186/s40851-017-0062-y

**Published:** 2017-02-12

**Authors:** Marcelo R. Sánchez-Villagra, Analía M. Forasiepi

**Affiliations:** 1 Paläontologisches Institut und Museum der Universität Zürich, Karl Schmid Strasse 4, Zürich, 8006 Switzerland; 2 IANIGLA, CCT-CONICET Mendoza, Av. Ruiz Leal s/n°, Mendoza, 5500 Argentina

**Keywords:** Ontogeny, Skull, Tegmen tympani, Auditory bulla, Entotympanic, Tubal element, *Dromiciops*, *Monodelphis*, *Macropus*, *Perameles*

## Abstract

An overview of the literature on the chondrocranium of marsupial mammals reveals a relative conservatism in shape and structures. We document the histological cranial anatomy of individuals representing *Monodelphis domestica*, *Dromiciops gliroides*, *Perameles* sp. and *Macropus eugenii*. The marsupial chondrocranium is generally characterized by the great breadth of the lamina basalis, absence of pila metoptica and large otic capsules. Its most anterior portion (cupula nasi anterior) is robust, and anterior to it there are well-developed tactile sensory structures, functionally important in the neonate. Investigations of ossification centers at and around the nasal septum are needed to trace the presence of certain bones (e.g., mesethmoid, parasphenoid) across marsupial taxa. In many adult marsupials, the tympanic floor is formed by at least three bones: alisphenoid (alisphenoid tympanic process), ectotympanic and petrosal (rostral and caudal tympanic processes); the squamosal also contributes in some diprotodontians. The presence of an entotympanic in marsupials has not been convincingly demonstrated. The tubal element surrounding the auditory tube in most marsupials is fibrous connective tissue rather than cartilage; the latter is the case in most placentals recorded to date. However, we detected fibrocartilage in a late juvenile of *Dromiciops*, and a similar tissue has been reported for *Tarsipes*. Contradictory reports on the presence of the tegmen tympani can be found in the literature. We describe a small tegmen tympani in *Macropus*. Several heterochronic shifts in the timing of development of the chondocranium and associated structures (e.g., nerves, muscles) and in the ossification sequence have been interpreted as largely being influenced by functional requirements related to the altriciality of the newborn marsupial during early postnatal life. Comparative studies of chondocranial development of mammals can benefit from a solid phylogenetic framework, research on non-classical model organisms, and integration with imaging and sectional data derived from computer-tomography.

“…in morphology general principles are founded on matters of quite intricate detail and require detail for their illustration.”

De Beer [1], page xxix.

## Background

The study of comparative anatomy has been dramatically enhanced in the last decades by the availability of three-dimensional, non-invasive imaging made possible by computed tomography (hereafter, CT scanning) [2–4]. Scanning offers a means to acquire high-quality information from areas of the skull that would otherwise be unavailable except via invasive techniques.

Studies based on CT scanning involve the documentation of sections, analogous to histological work. In this regard, CT scanning is a very helpful guide, permitting quick identification of structures and the reconstruction of soft-tissues without the effort needed to produce well-procesed and stained serial sections [5]. High-resolution CT scanning, together with new methods of staining, have been used to generate three-dimensional images of organs and soft tissues, which could potentially have a significant impact on studies of organogenesis and anatomy [6, 7]. However, the precise level of anatomical resolution which may be attained by histological serial sectioning cannot yet be matched by CT scanning. Thanks to color differentiation, serial sections can provide detailed information about cartilage, membranes, relationships of blood vessels and nerves, and different tissues and organs, in some cases leading to the discovery of cladistically diagnostic features [8–12]. Histological serial sections can also provide information on the nature of the tissues, giving insights into their origin, development and distribution of mechanical strain [13].

**Fig. 1:**
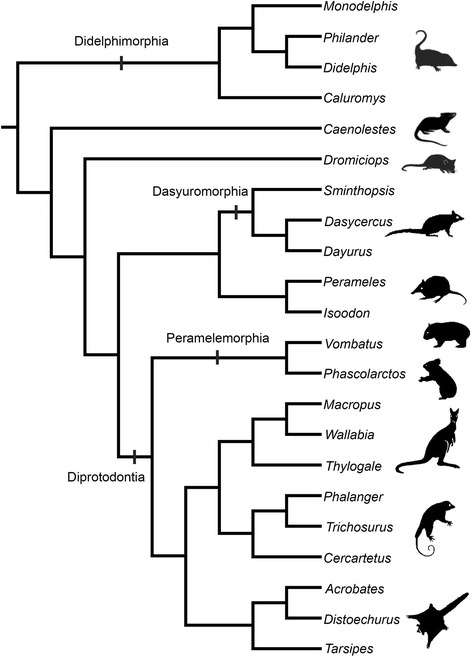
Phylogeny of main species discussed in the text, representatives of several major groups of extant marsupials, including Didelphimorphia, Microbiotheria, Peramelemorphia and Diprotodontia. Phylogeny of diprotodontians is after Meredith et al. [211], of dasyuromorphians is after Westerman et al. [212]; major relationships among groups based on Gallus et al. [213] and Beck et al. [214]. Animal outlines modified from Horovitz and Sánchez-Villagra [102] and from Gallus et al. [213]

The objective of this contribution is to provide a reference on the sectional anatomy of the developing marsupial head, summarize major features of the anatomy of the marsupial chondrocranium, and discuss some controversial issues concerning the ethmoidal, orbitotemporal and basicranial regions. We document species that represent several major groups of extant marsupials, including Didelphimorphia, Microbiotheria, Peramelemorphia and Diprotodontia (Fig. 1).

### Marsupial evolution, cranial anatomy and development

Marsupialia is the crown group that includes the common ancestor of all extant marsupials and their descendants. Metatheria is the most inclusive group of mammals more closely related to opossums and their fossil relatives than to placentals [14]. Metatheria encompasses stem marsupials going back to perhaps Jurassic times [15].

Traditionally, marsupials, in particular opossums (didelphids), have been taken as models to investigate early mammalian evolution [16–19]. Several plesiomorphic features retained in the musculoskeletal system resulted in the interpretation of opossums as models for understanding the therian last common ancestor. However, this does not imply that their anatomy is lacking in complexity [20]. *Monodelphis* is a didelphid of growing importance in biomedical research, and one that has been used in evolutionary studies of mammalian development [21–23]. *Dromiciops* is a key taxon to understand issues within marsupial phylogeny, representing a member of an otherwise Australasian clade (Fig. 1).

Several aspects of the adult cranial anatomy of marsupials have been treated since the late 19th century (e.g., [9, 17, 24–37]). These works are based mostly on the macroscopic examination of macerated skulls, including that of Wible [30] on the skull of *Monodelphis brevicaudata*, which clarifies terminological issues and establishes a clear reference to didelphid adult anatomy (see also [38]).

The architecture of the embryonic marsupial cranium has been interpreted as largely influenced by functional requirements related to a particular mode of reproduction [39]. It is assumed that the cranium of the altricial newborn marsupial must be able to withstand the mechanical strain of sucking at the teat, from which it is suspended within the marsupium [40–43]. Heterochronic shifts have been detected towards the extreme precocity of ossification of the bones of the snout (e.g. premaxilla, maxilla, palatine, dentary), particularly the palate [1] and the back of the skull (e.g., exoccipital) [44] to support active suckling. With respect to the chondrocranium, comparisons with monotremes, which do not suckle at birth, are needed [12, 45] as to establish the polarity of many traits and test some of the claims on a novel functional significance for several marsupial traits.

The chondrocranium consists of numerous individual chondrification centers that appear at different times and fuse, forming a single structure that forms the earliest phase of the developing fetal skull. Most of the chondrocranium is replaced by ossifications; however, some parts become resorbed in later ontogenetic stages while others persist into the adult stage (e.g., nasal cartilages). The skull has different ossification modes; it is completed via direct (intramembranous) and indirect (perichondral and endochondral) osteogenesis. These processes operating in the formation of the different kinds of bones and their histology, ontogenetic and phylogenetic history, have been discussed comprehensively by Hall [13] and Padian and Lamm [46]. Intramembranous osteogenesis, also called periosteal or subperiosteal osteogenesis [13], represents the conversion of a fibrous mesenchymal precursor directly into bone, the result of which is usually called “dermal” bone. Perichondral and endochondral osteogenesis require an intermediate cartilaginous precursor before bone deposition. This sequence produces endochondral bones, also called “replacement” bones. Appositional bone (“Zuwachsknochen”) does not have a cartilaginous precursor [47] and thus resembles dermal bone, although it grows by apposition on a cartilaginous core.

### Developing head anatomy—the chondrocranium

Much of the literature on cranial marsupial development is from the first part of the 20th century. What makes it somewhat difficult to access is not so much the fact that most of it was published in German, but rather that it is highly descriptive in nature, without what we would understand today as a clear phylogenetic underpinning, and with a plethora of terms that tend to describe single or only a few stages. In what follows, our descriptions suffer from these two limitations by necessity. In most cases the kind of information necessary for explicit analytical quantification of character evolution is entirely missing (e.g., [48]).

Broom [49–52] studied several aspects of the marsupial chondrocranium. There are also detailed studies on the common opossum *Didelphis marsupialis* by Toeplitz [53], the bare-tailed woolly opossum *Caluromys philander* by Denison and Terry [54], the eastern quoll *Dasyurus viverrinus* by Broom [51], and the long-nosed and the short nose bandicoot *Perameles nasuta* and *P. obesula* by Cords [55] and Esdaile [56], respectively. Maier presented a series of contributions [31, 41, 42, 57–59] richly informative on the short-tailed opossum *Monodelphis domestica* and other marsupials. Several of his students at the Universities of Tübingen and Frankfurt wrote theses on other species, including Klutzny [60] on the wombat *Vombatus ursinus*, Müller [61] on the swamp wallaby *Wallabia rufogrisea*, and Schmelzle [62] on the tammar wallaby *Macropus eugenii*. Clark and Smith [63] and Smith [64] presented a great deal of documentation on the histological cranial anatomy of *Monodelphis domestica* and *Macropus eugenii*.

In this contribution, much of the data on developmental cranial anatomy presented in the doctoral thesis of Sánchez-Villagra [65] are presented for the first time. The doctoral dissertations of Wible [9] and of Aplin [66] are major contributions to the subject. The information on marsupials in Wible [9] contains copious unpublished details, which add to his many contributions on cranial anatomy [67, 68]. The doctoral thesis of Aplin [66] includes thorough documentation, description and comparisons of the basicranial region of diprotodontian marsupials in which many taxa are examined for this first time with histological series.

Comparisons with marsupials have been made in studies on the chondrocranium and osteocranial development of placentals (e.g., [8, 69–71]) and monotremes [45, 72]; see overviews in de Beer [1] and Moore [73], and general revisions on ontogeny illustrated under a broad phylogenetic context [74].

The temporal window of development examined in this paper is one that can be best described as “perinatal”, meaning the time around birth. This is a critical time in organogenesis [75], traditionally omitted by embryologists concerned with earlier stages of development (e.g., [76–78]), or by those interested in growth, for which a morphometric study of postnatal stages is usually applied (e.g., [79, 80]). These boundaries are increasingly overstepped by studies that examine growth trajectories across much of ontogeny [81–83], or by those concerned with organogenesis and sequence in development [84]. As stated by Maier ([75]:60) regarding data on perinatal stages, these “not only narrow an existing gap of morphological knowledge, but they refer to a phase of life which is very peculiar and specialized in mammalian life history”. Given the wide range of altricial to precocial development at birth [85], the latter is not a reliable stage of comparison across mammals, so the term “perinatal” is used here in a broad sense.

It is important to consider the timeframe in which the chondrocranium, the focus of much of this work, is formed, but has not yet begun to differentiate into bone. That stage is one that has been called the “critical period” or *stadium optimum*. In the context of a description of two marsupial embryos, Beard [86] stated: “The ‘critical period’ in a morphological sense is that epoch of the development when all the parts of the organism are first present as the foundations or ‘Anlagen’ of all the organs; it is that state when epigenesis is ended, and evolution or unfolding is beginning; it is that point where the individuality of the organism is first attained, when it has acquired a something setting it down as the embryo of some particular form, and when it is first beginning to resemble its progenitors”. In reality, the difficulty in comparative studies of the chondrocranium lies in the fact that there is no objective way to identify comparable stages [87, 88]. A series including several stages is thus desirable, but acquiring the necessary specimens is time-consuming. That is why some authors have followed the strategy of focusing on regions of the chondrocranium for extensive comparisons including developmental series of different species [48, 89, 90]. The perinatal time is arguably the most important period for understanding critical aspects of the developmental anatomy of the mammalian skull and the homology of its various components [8, 71, 91].

## Materials and methods

**Fig. 2:**
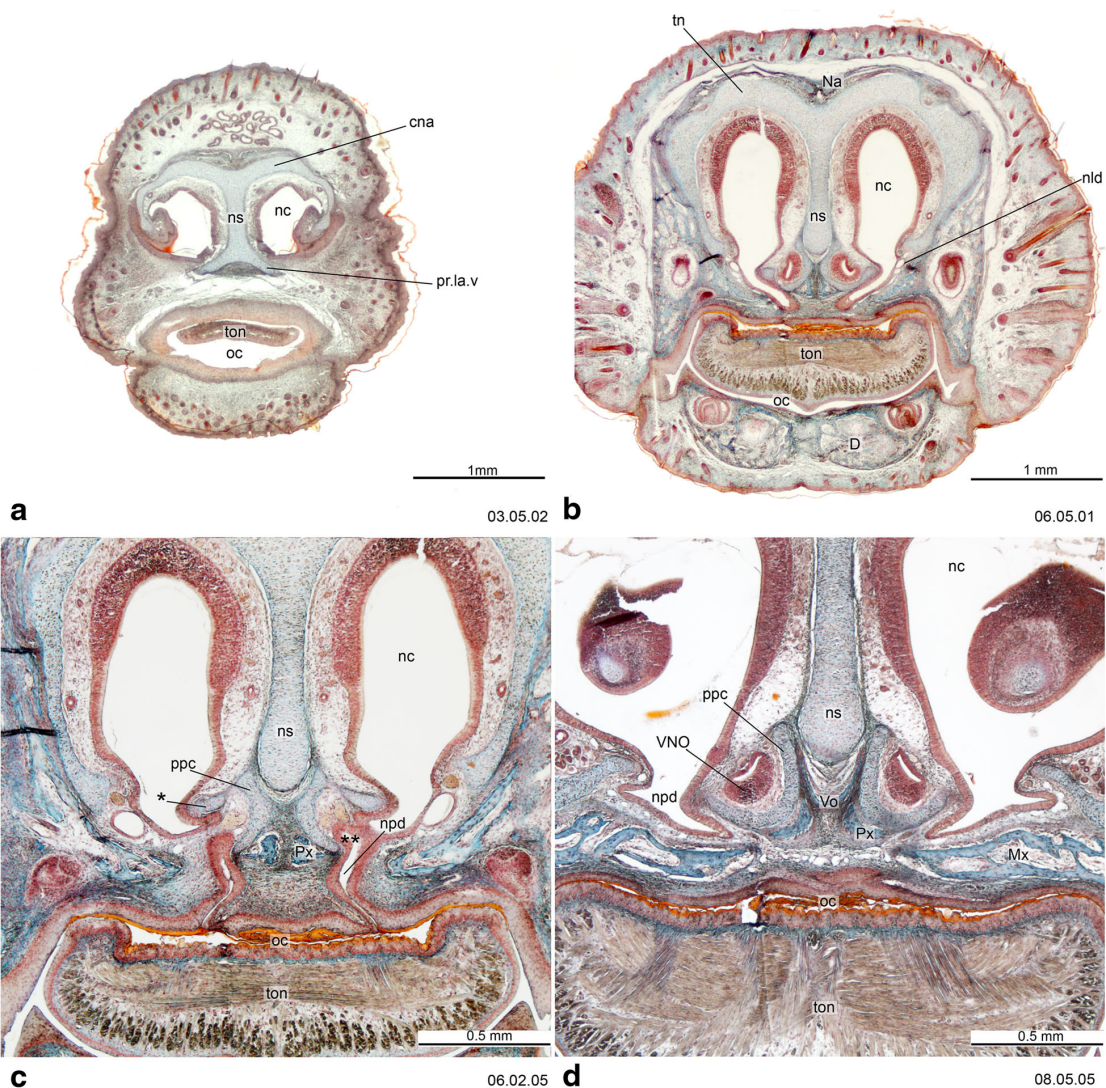
Cross-sections of *Monodelphis domestica* (PND 12, HL 8.5 mm) at the ethmoidal region, with detail of the vomeronasal complex. The *asterisk* (*) in **c** indicates the “outer bar” [49] or “fibula reuniens” [215] which is a lateral portion of the paraseptal cartilage. The *double asterisk* (**) in **c** indicates the lumen of VNO opening in the nasopalatine duct. Numbers of the histological serial sections are indicated at the *bottom left* of each figure

**Fig. 3:**
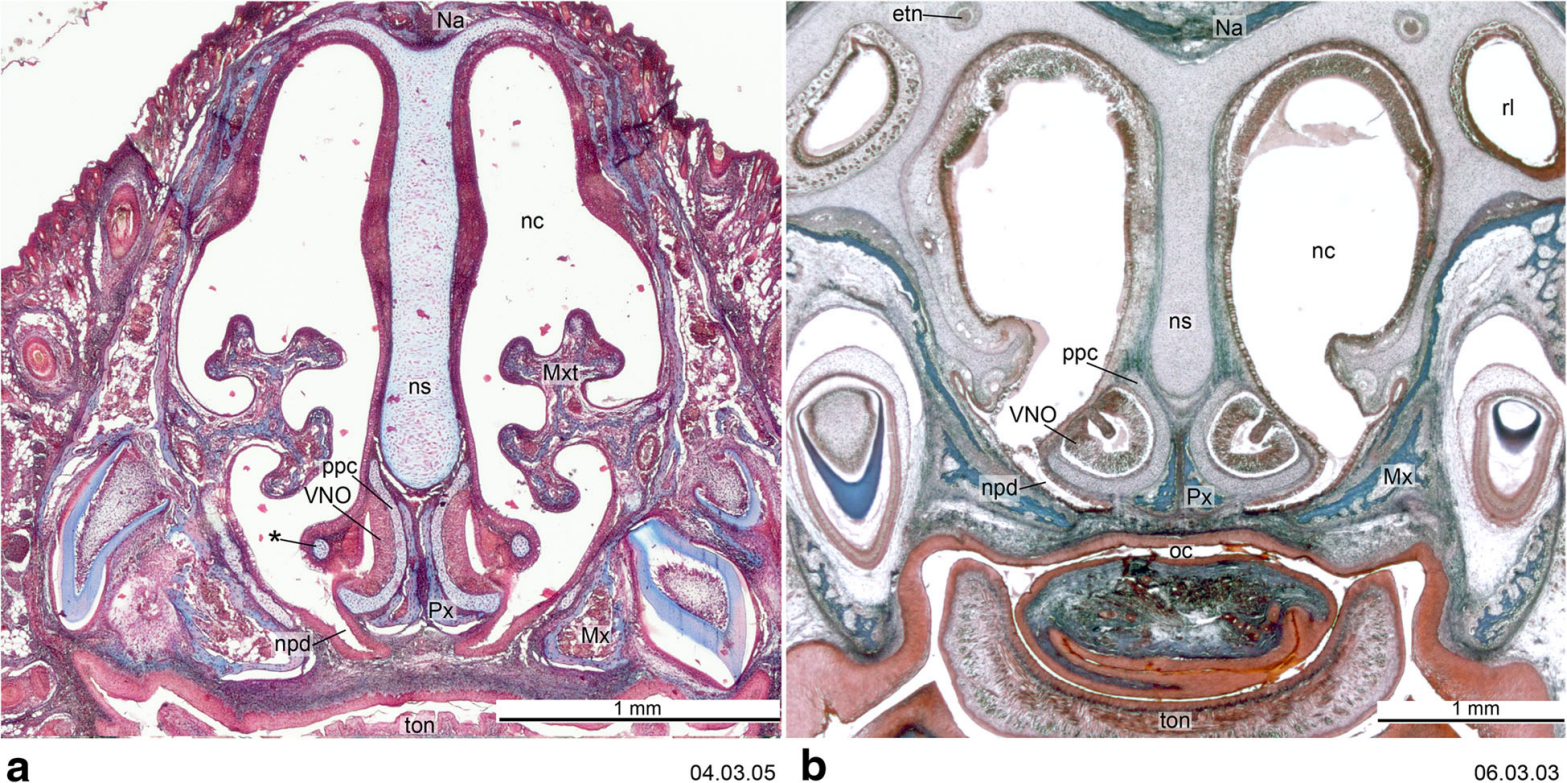
Cross-section of the vomeronasal complex of *Dromiciops gliroides*, ZIUT, HL 19 mm (**a**) and *Perameles* sp., ZIUT, HL 17.5 mm. (**b**) The *asterisk* (*) in **a** indicates the “outer bar”. Numbers of the histological serial sections are indicated at the bottom right of each figure. Numbers ascend in caudal direction

**Fig. 4:**
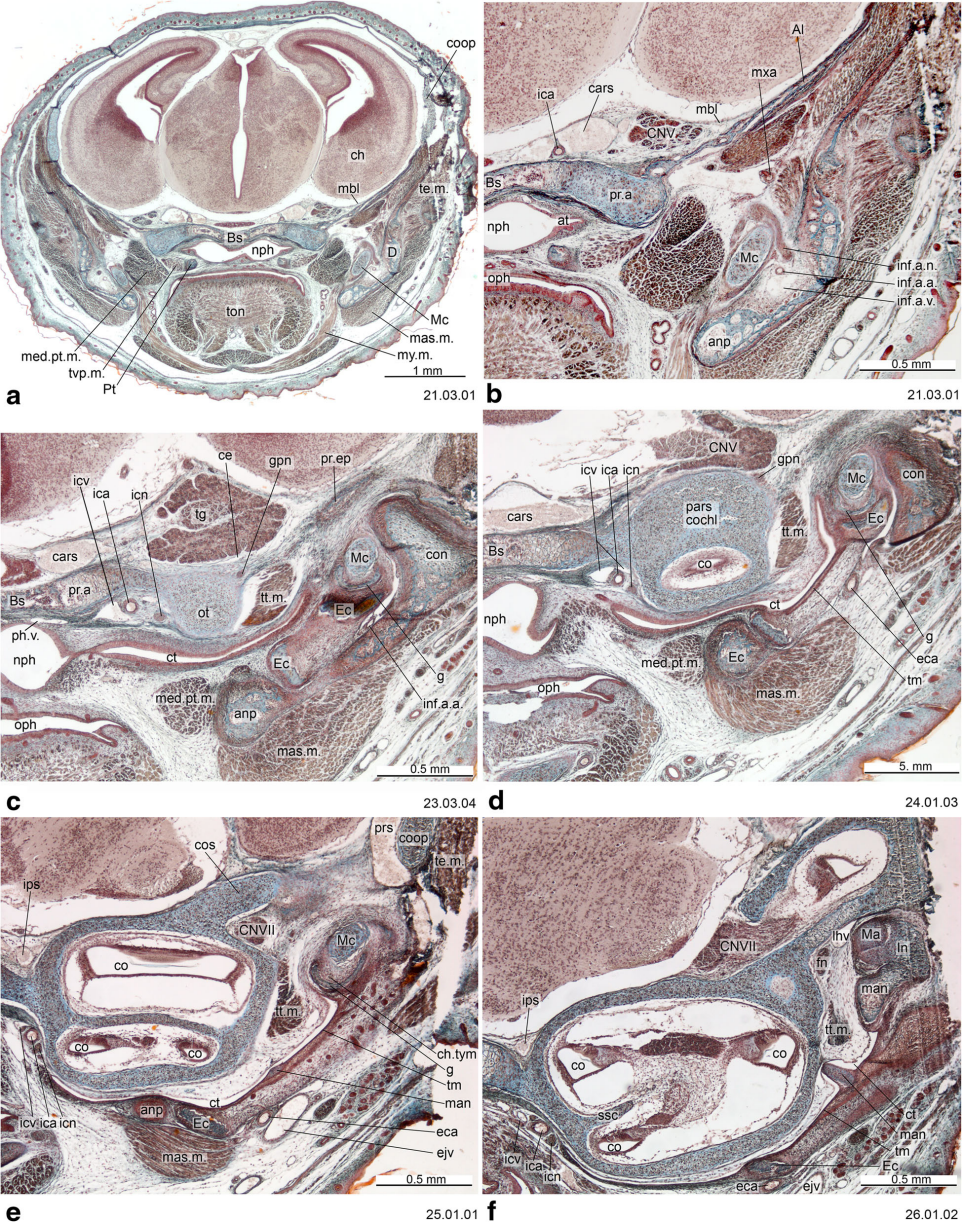
Cross-section of *Monodelphis domestica* (PND 12, HL 8.5 mm) at the ear region. Numbers of the histological serial sections are indicated at the bottom right of each figure. Numbers ascend in caudal direction

**Fig. 5:**
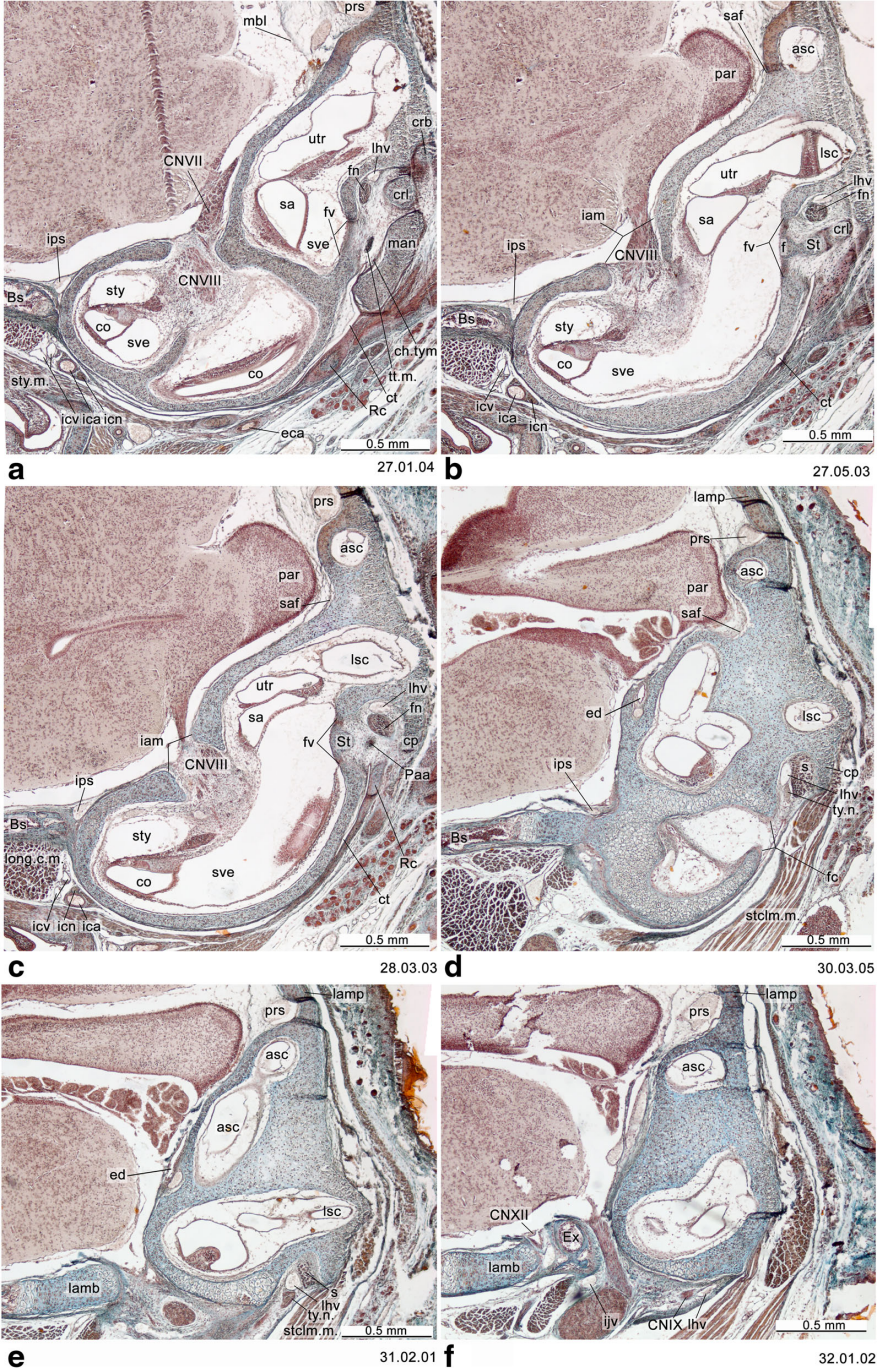
Cross-section of *Monodelphis domestica* (PND 12, HL 8.5 mm) at the level of the otic capsule. Numbers of the histological serial sections are indicated at the bottom right of each figure. Numbers ascend in caudal direction

**Fig. 6:**
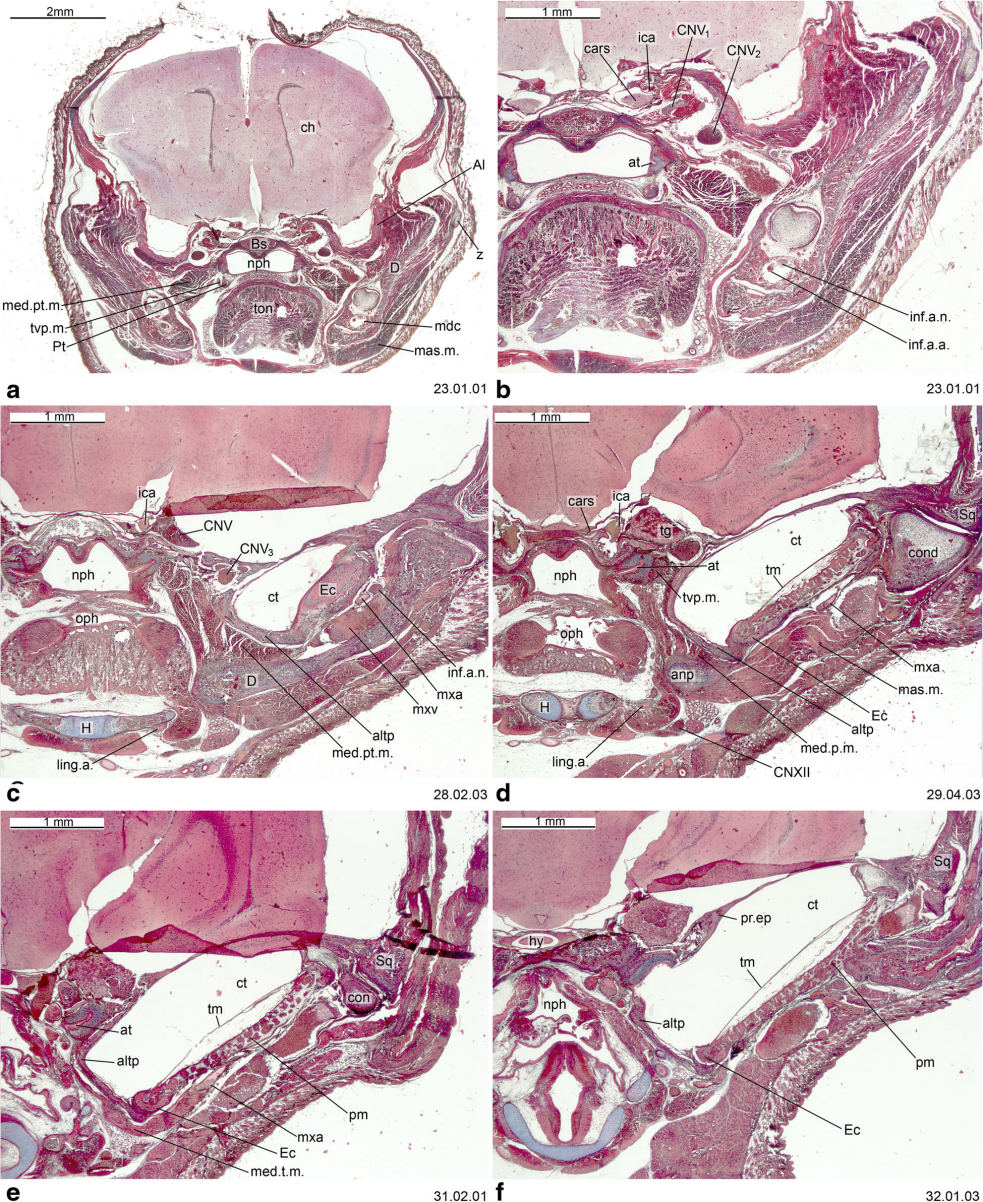
Cross-section of *Dromiciops gliroides* (ZIUT, HL 19 mm) at the level of the ear region. Numbers of the histological serial sections are indicated at the bottom right of each figure. Numbers ascend in caudal direction

**Fig. 7:**
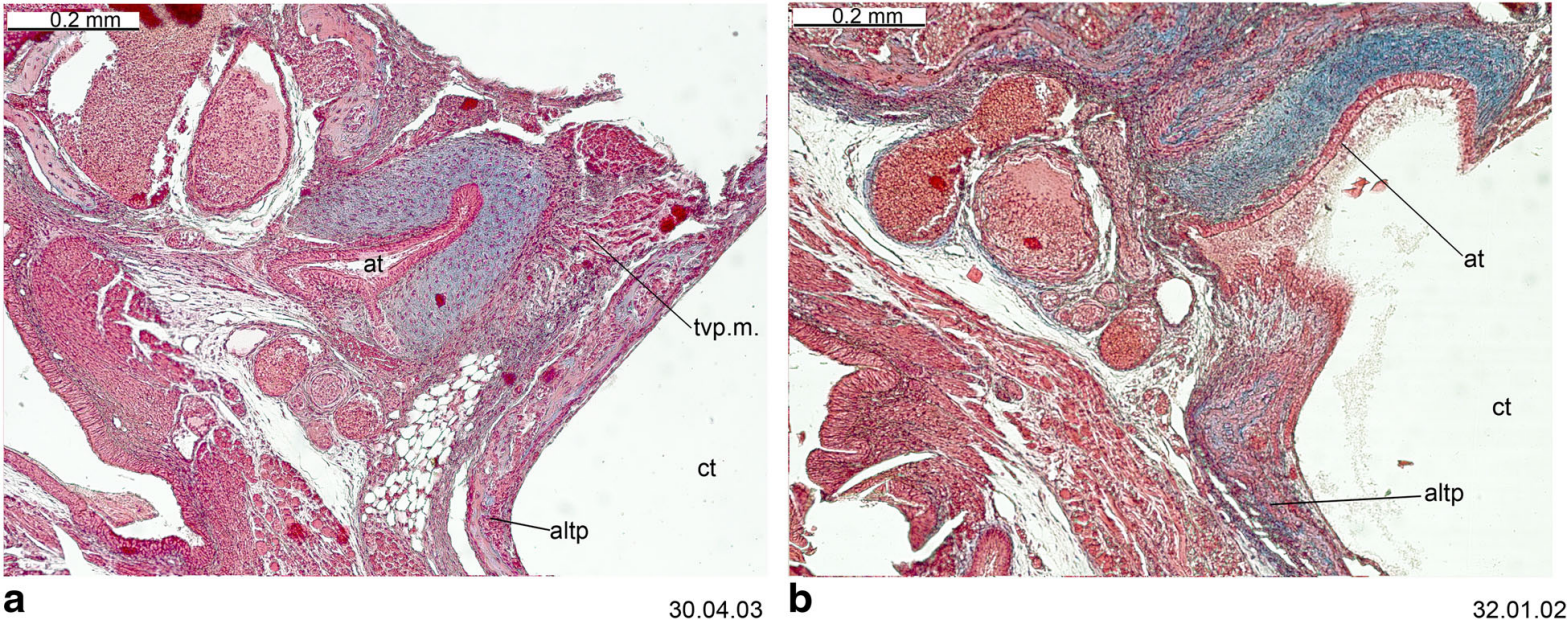
Cross-section of *Dromiciops gliroides* (ZIUT, HL 19 mm) with the fibrocartilaginous element of the auditory tube. Numbers of the histological serial sections are indicated at the bottom right of each figure

**Fig. 8:**
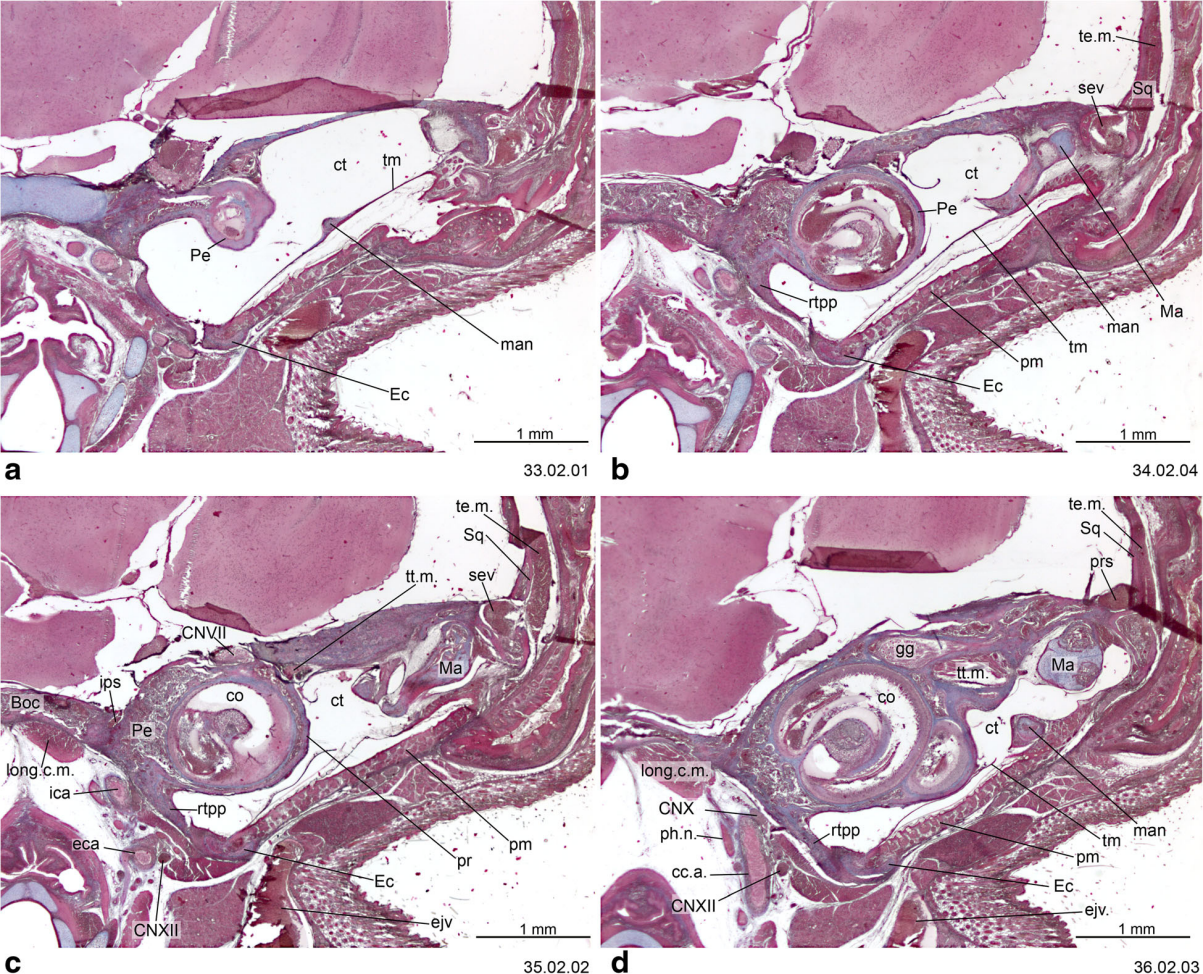
Cross-section of *Dromiciops gliroides* (ZIUT, HL 19 mm) at the level of the rostral part of the petrosal (pars cochlearis). Note the extensive contribution of the rtpp to the auditory capsule. Numbers of the histological serial sections are indicated at the bottom right of each figure. Numbers ascend in caudal direction

**Fig. 9:**
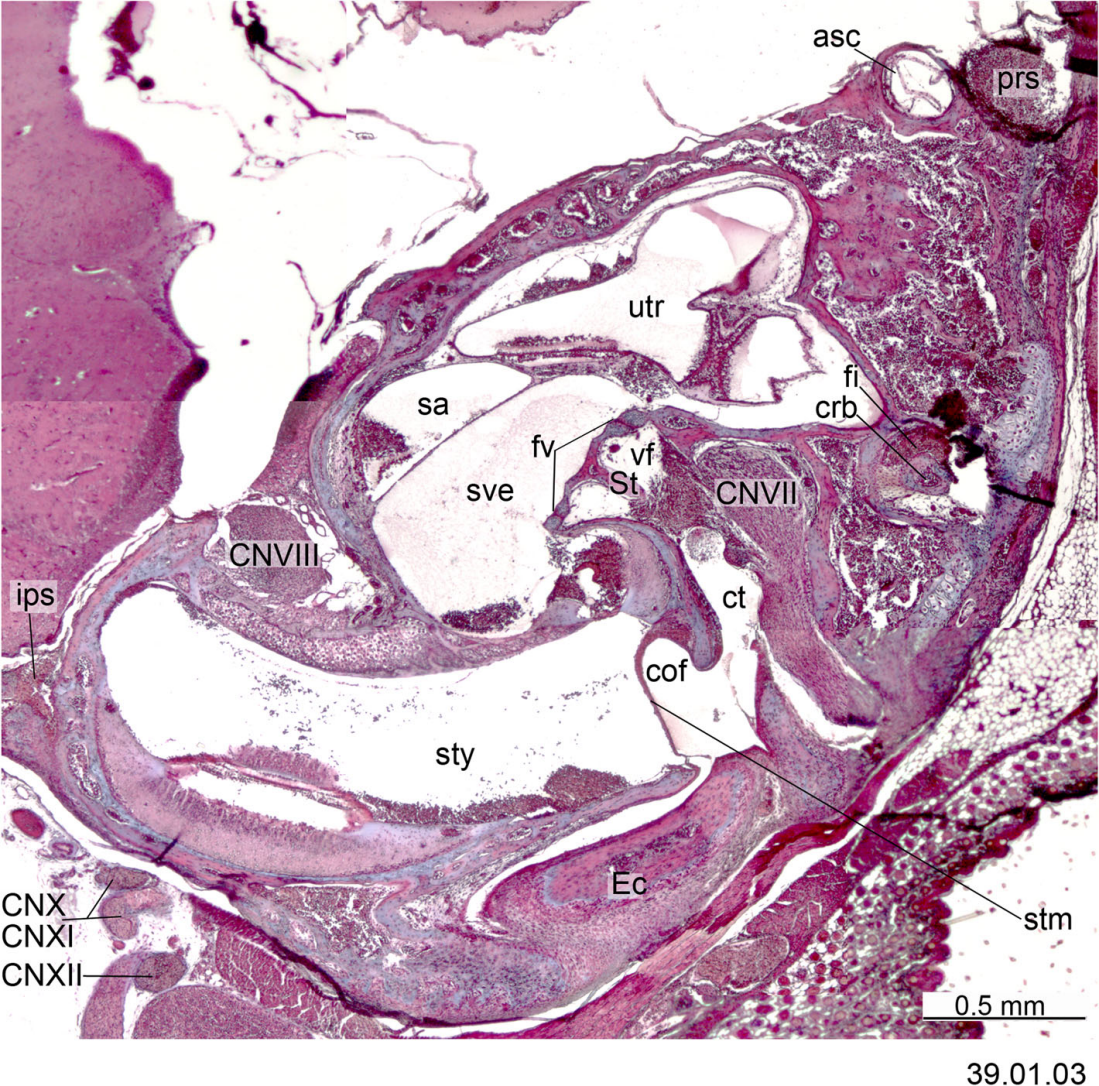
Cross-section of the petrosal of *Dromiciops gliroides* (ZIUT, HL 19 mm). Number of the histological serial section is indicated at the bottom right

**Fig. 10:**
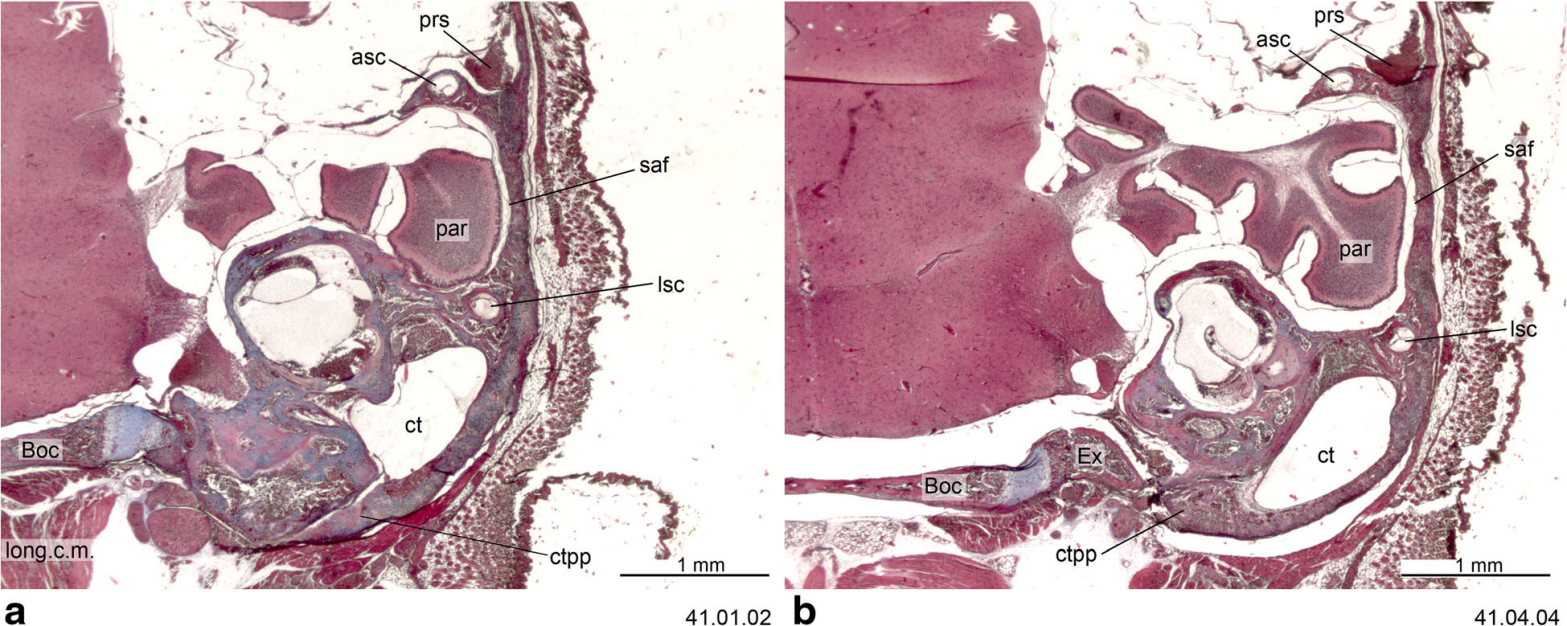
Cross-section of *Dromiciops gliroides* (ZIUT, HL 19 mm) at the level of the caudal part the petrosal (pars canalicularis). Note the extensive contribution of the ctpp to the tympanic floor. Numbers of the histological serial sections are indicated at the bottom right of each figure. Numbers ascend in caudal direction

**Fig. 11:**
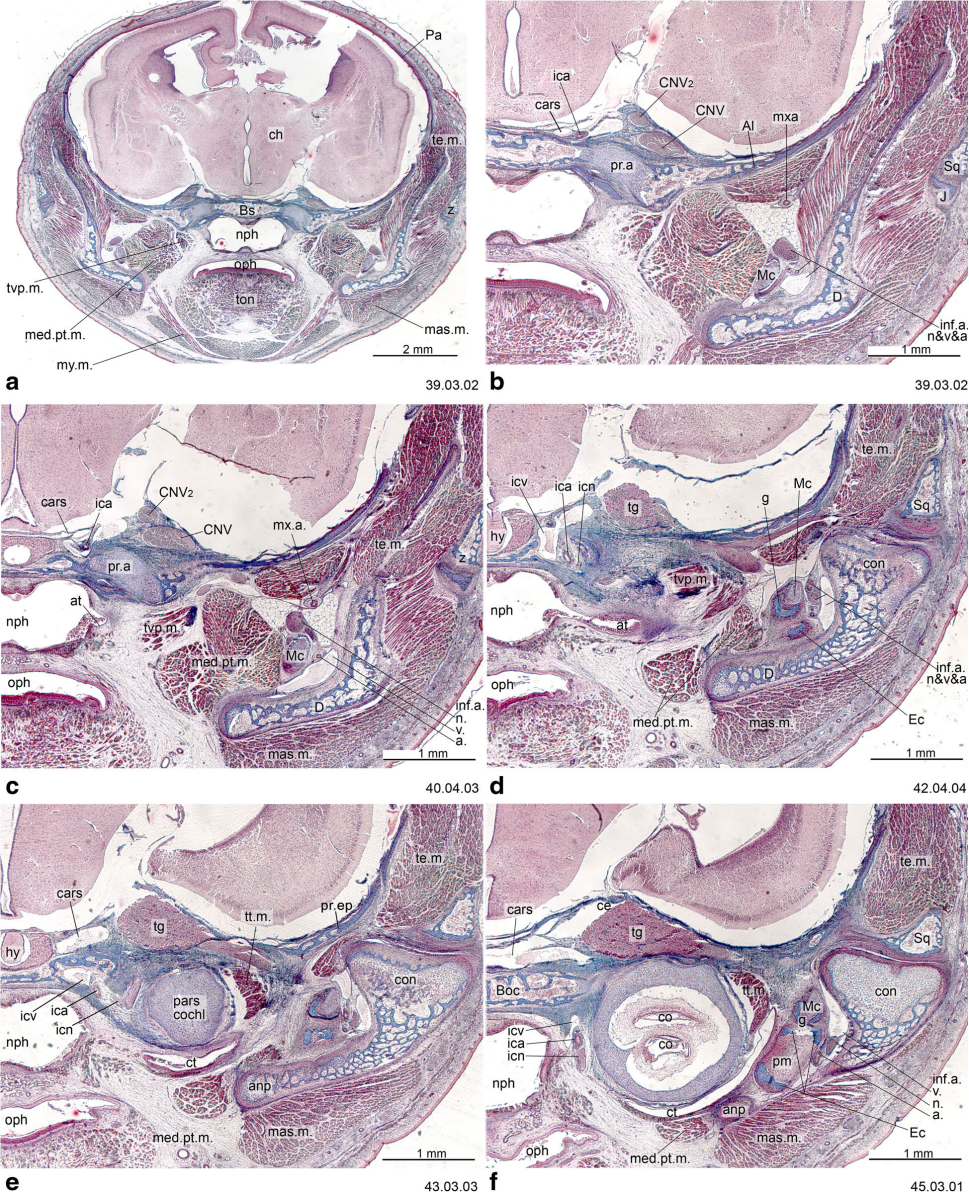
Cross-section of *Perameles* sp. (ZIUT, HL 17.5 mm) at the level of the ear region. Numbers of the histological serial sections are indicated at the bottom right of each figure. Numbers ascend in caudal direction

**Fig. 12:**
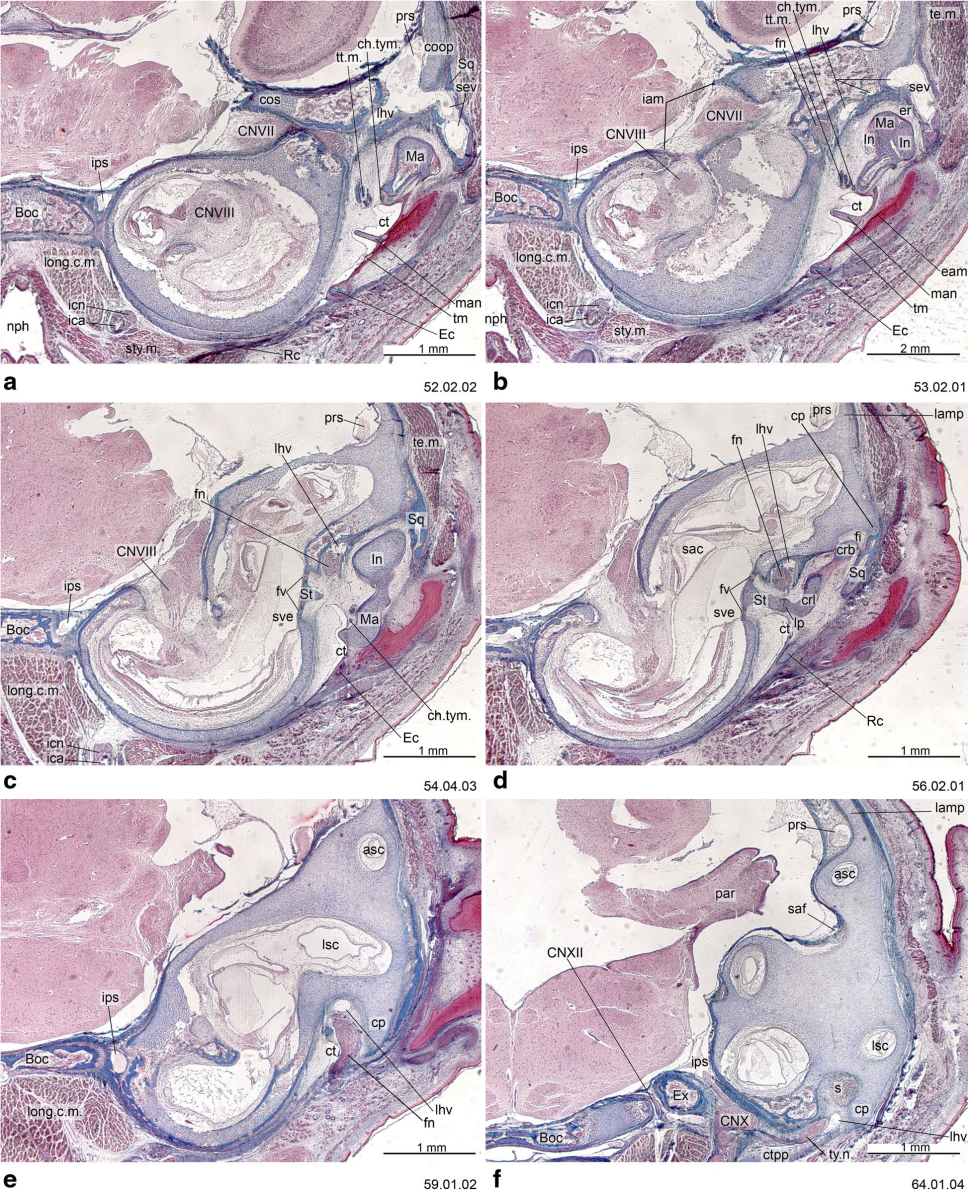
Cross-section of *Perameles* sp. (ZIUT, HL 17.5 mm) at the level of the otic capsule. In section 53.02.01, the lateral head vein is inside the prootic canal. The lateral head vein is the boundary for the prootic sinus and the sphenoparietal emissary vein, and it is retained in some adult marsupials [68]. Numbers of the histological serial sections are indicated at the bottom right of each figure. Numbers ascend in caudal direction

**Fig. 13:**
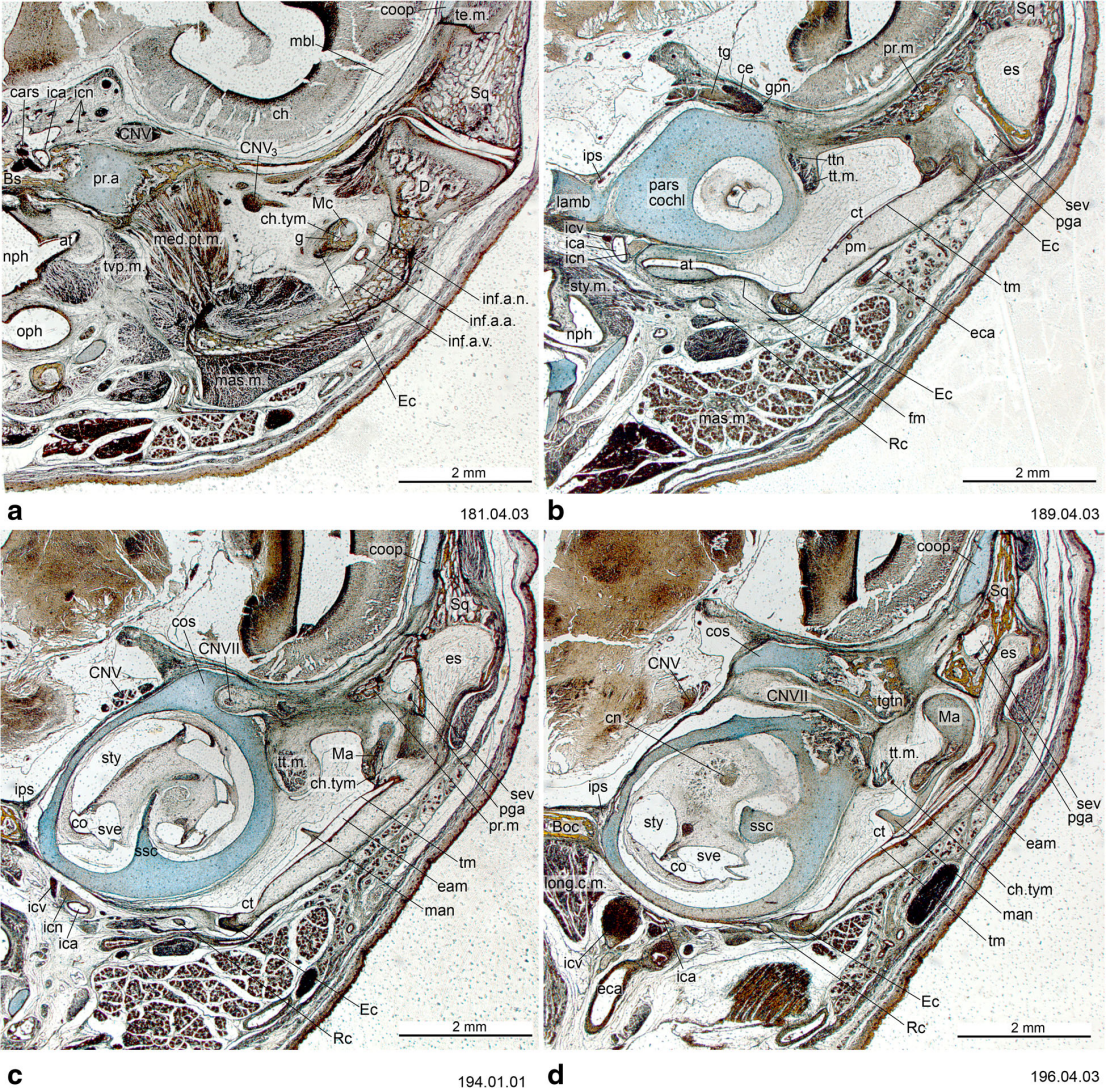
Cross-sections of *Macropus eugenii* (ZIUT, HL 29 mm) at the level of the ear region. Note the large epitympanic recess in **b** and **c**. Numbers of the histological serial sections are indicated at the bottom right of each figure. Numbers ascend in caudal direction

**Fig. 14:**
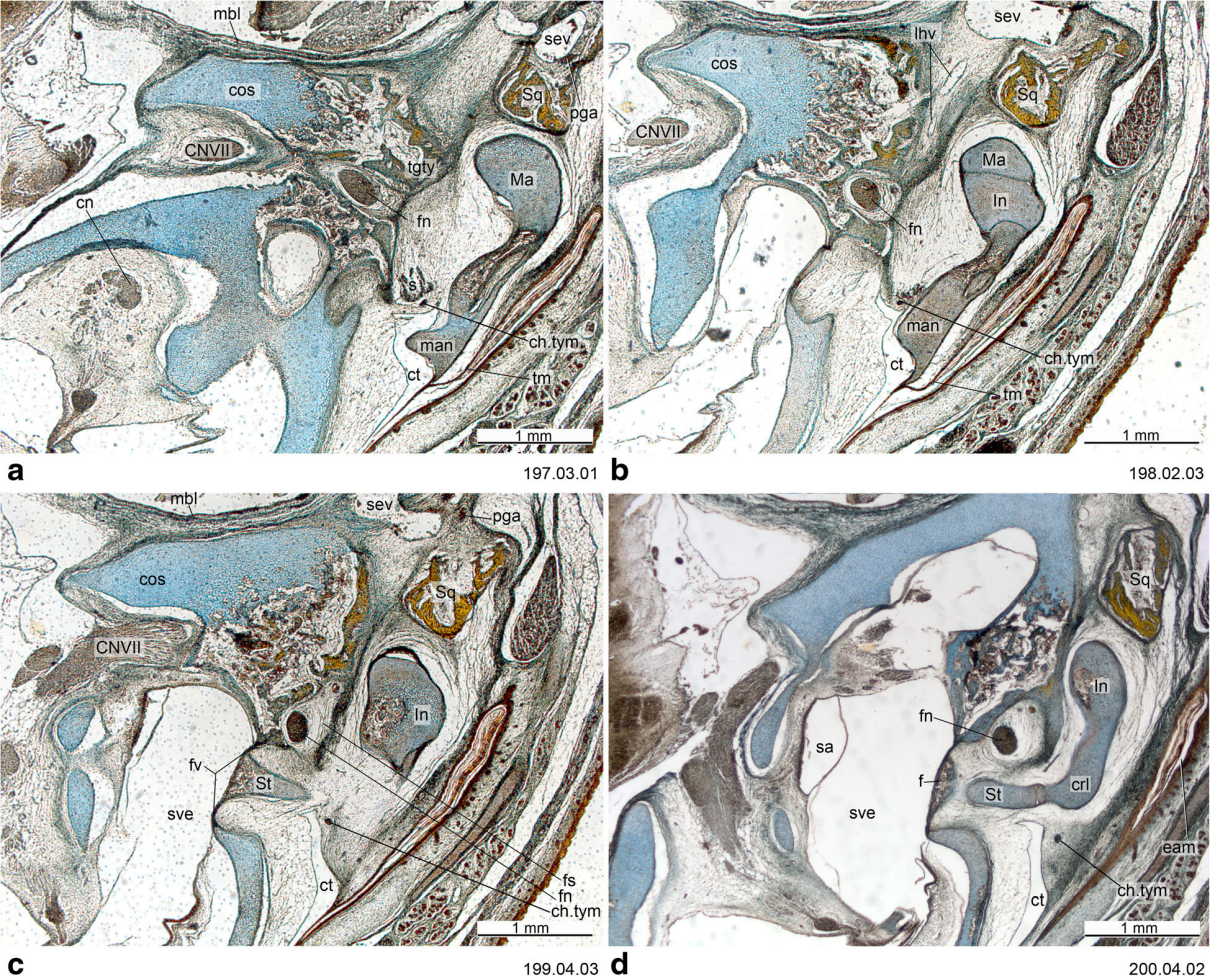
Cross-section of *Macropus eugenii* (ZIUT, HL 29 mm) at the level of the petrosal. In section 198.02.03, the lateral head vein is in the prootic canal. Numbers of the histological serial sections are indicated at the bottom right of each figure. Numbers ascend in caudal direction

**Fig. 15:**
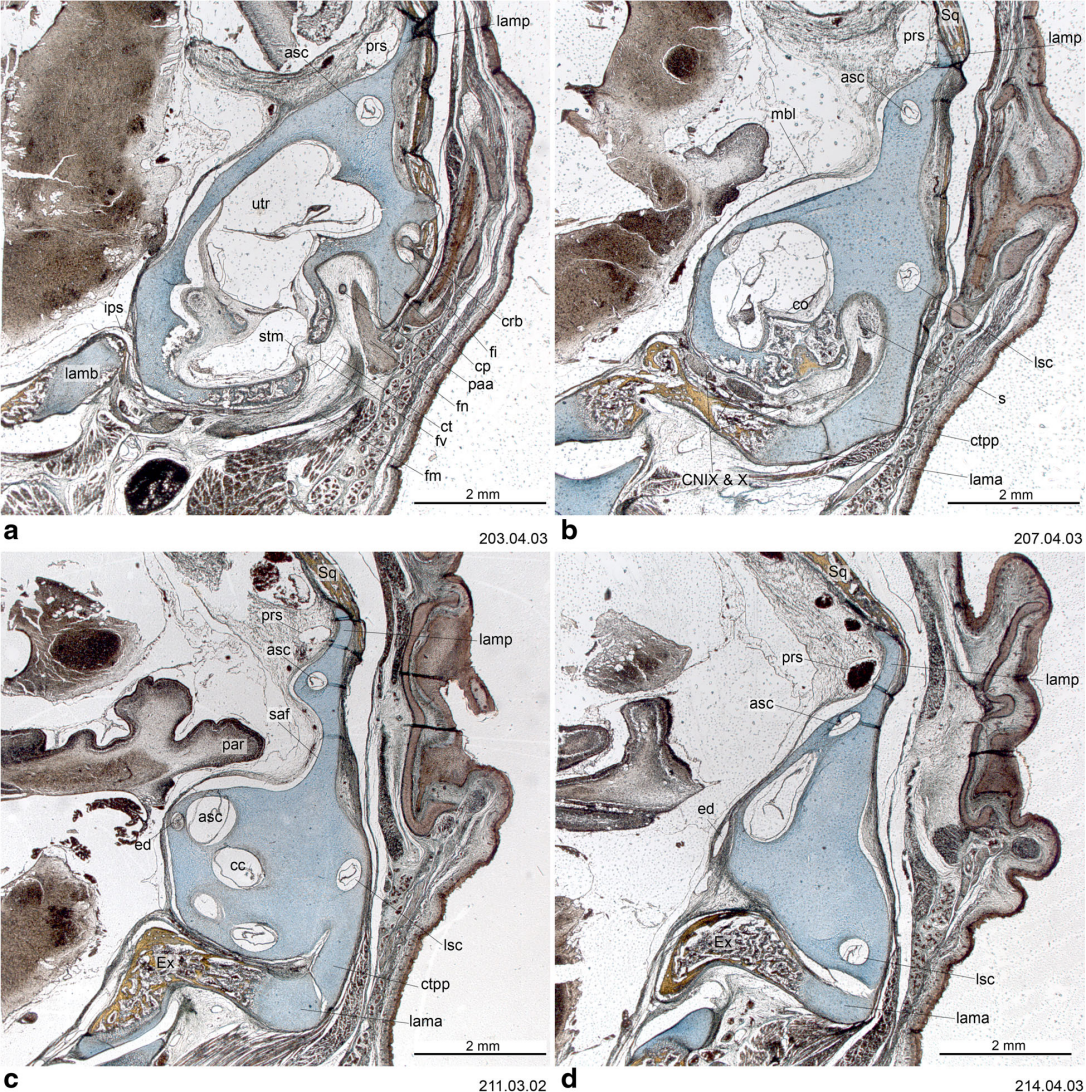
Cross-section of *Macropus eugenii* (ZIUT, HL 29 mm) at the level of the caudal part the petrosal (pars canalicularis). Note the extensive ctpp (**b** and **c**) and the partial contribution to the floor of the tympanic cavity. Numbers of the histological serial sections are indicated at the bottom right of each figure. Numbers ascend in caudal direction

**Fig. 16:**
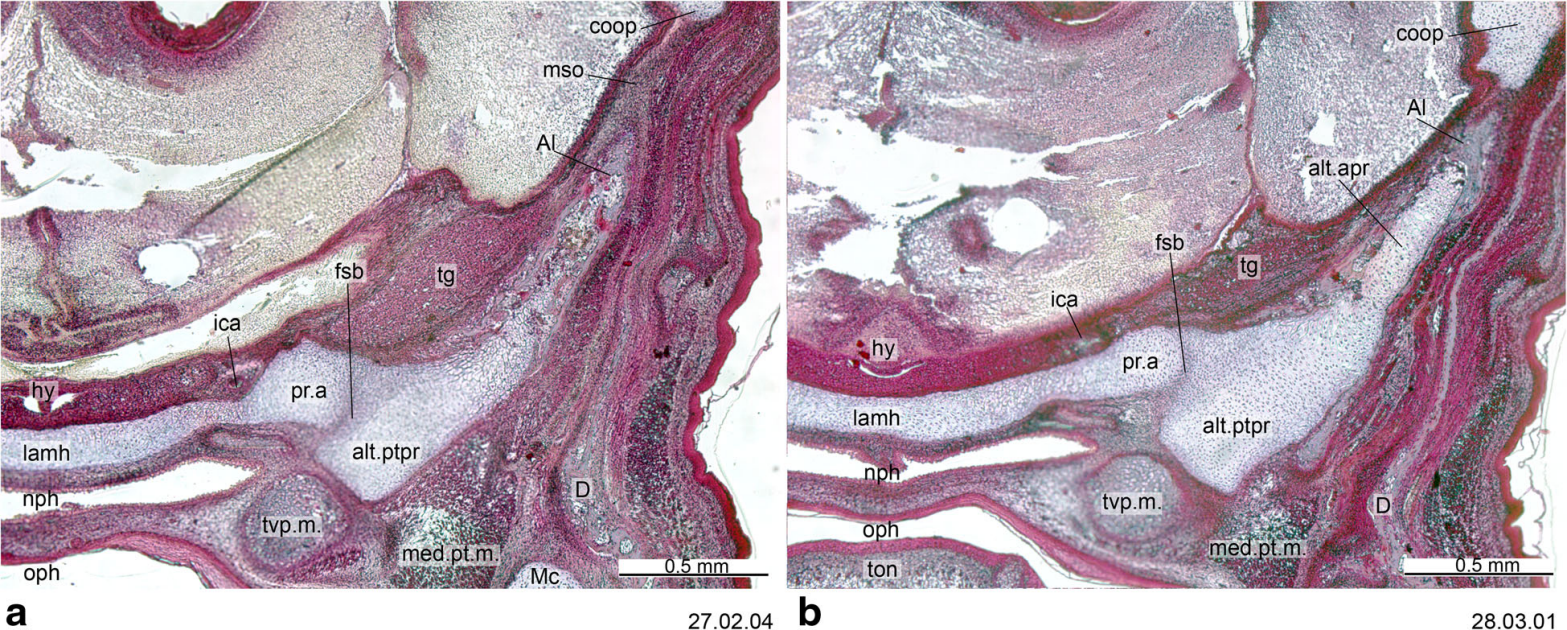
Cross-section of *Thylogale billardierii* (ZIUT, HL 13 mm) at the level of the hypophysis. The chondrocranial braincase wall if formed by the lamina ascendens of the ala temporalis and the membrana sphenoobturatoria, which in this specimen have started ossification. Numbers of the histological serial sections are indicated at the bottom right of each figure. Numbers ascend in caudal direction

Figures 2, 3, 4, 5, 6, 7, 8, 9, 10, 11, 12, 13, 14, 15 and 16 illustrate the histological cranial anatomy of individuals representing four species: *Monodelphis domestica* postnatal day (PND) 12 (head length HL 8.5 mm), *Dromiciops gliroides*, ZIUT (HL 19 mm), *Perameles sp*. ZIUT (HL 17.5 mm), *Macropus eugenii* ZIUT (HL 29 mm), and *Thylogale billardierii* (HL 13 mm). Histological frontal sections were photographically recorded with a stereoscopic microscope (Leica MZ 16®) under natural light. Images were captured with a Leica DFC 420 C® digital camera and contrast was enhanced with Adobe Photoshop®. Table 1 lists the abbreviations used throughout the figures of this paper. The serial sections studied are deposited at the Universität Tübingen, collection of W. Maier from the former Zoologisches Institut, Germany (ZIUT)—some in long-term loan to the senior author’s laboratory at the University of Zurich; Duke University Comparative Embryology Collection, currently housed at the Evolutionary Anthropology Department, Durham NC USA (DUCEC). Anatomical nomenclature follows mainly MacPhee [8] and Maier [41, 42, 57], but as noted, terminology for the chondrocranium is diverse [12, 70]. Other useful sources of definitions and clarifications of skull anatomy are Wible and Rougier [92] and Mead and Fordyce [93]. The nomenclature of the ethmoidal region was discussed by Maier ([42], fig. 12.11), Freyer [94], and Rowe et al. [95].

**Fig. 17:**
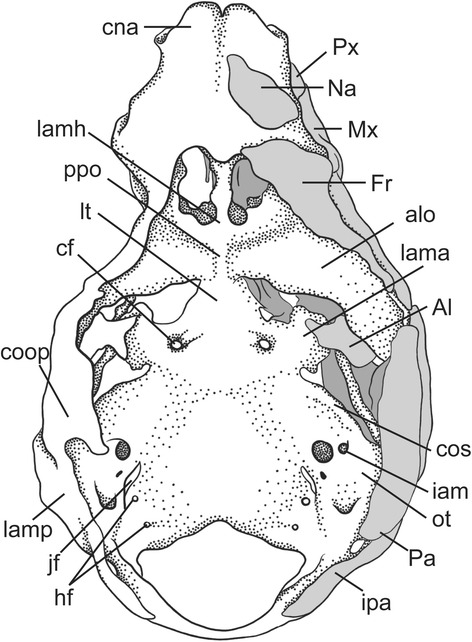
Dorsal view of the chondrocranium of a diprotodontian marsupial, the wallaby *Wallabia rufogrisea* (CRL 37 mm) modified from Müller [61]. On the *right side*, depicted also the dermal bones documented at this stage

**Fig. 18:**
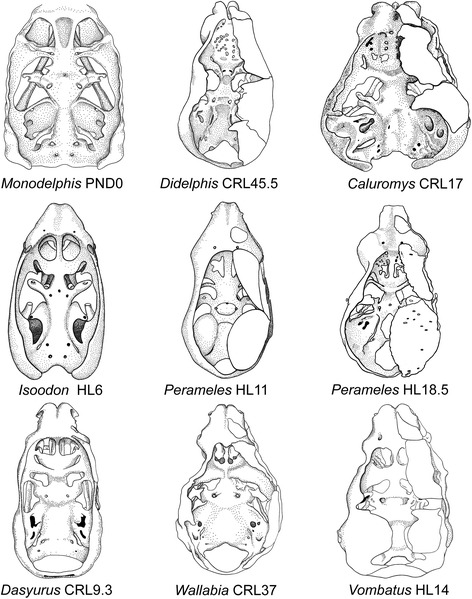
Dorsal view of chondrocrania of marsupials at different stages of development. On the *right side*, depicted also the dermal bones documented at the respective stage. Drawings were modified from the cited sources. Measurements are in millimeters. Depicted are models of the short-tailed opossum *Monodelphis domestica* by Maier [57], the opossum *Didelphis marsupialis* by Toeplitz [53], the bare-tailed woolly opossum *Caluromys philander* by Denison and Terry [54], the short nose bandicoot *Isoodon obesulus* by Esdaile [56], the long-nosed bandicoot *Perameles nasuta* by Cords [55], the eastern quoll *Dasyurus viverrinus* by Broom [51], the wombat *Vombatus ursinus* by Klutzny [60], and the wallaby *Wallabia rufogrisea* by Müller [61]

**Table 1:** List of the the abbreviations used throughout the figures of this paper

a., artery Al, alisphenoid alo, ala orbitalis alt.ptpr, ala temporalis processus ptegygoideus alt.apr, ala temporalis, processus ascendens altp, alisphenoid tympanic process anp, angular process of dentary asc, anterior semicircular canal at, auditory tube Boc, basioccipital Bs, basisphenoid cars, cavernous sinus cc, crus commune cc.a., common carotid artery ce, cavum epiptericum cf, carotid foramen ch, cerebral hemisphere ch.tym, chorda tympany n. (CN VII) cn, cochlear n. (CN VIII) cna, cupula nasi anterior CN V, trigeminal n. CN V_1_, ophthalmic branch of trigeminal n. CN V_2_, maxillary branch of trigeminal n. CN V_3_, mandibular branch of trigeminal n. CN VII, facial n. CN VIII, vestibulocochlear n. CN IX, glossopharyngeal n. CN X, vagus n. CN XI, accessory n. CN XII, hypoglossal n. co, cochlear duct cof, cochlear fossula (= fossula fenestra cochleae) con, dentary condyle coop, commisura orbitoparietalis cos, commisura suprafacialis cp, crista parotica crb, crus breve of incus crl, crus longum of incus ct, cavum tympani ctpp, caudal tympanic process of petrosal D, dentary eam, external acoustic meatus Ec, ectotympanic eca, external carotid a. ed, endolymphatic duct ejv, external jugular vein er, epitympanic recess es, epitympanic sinus etn, ethmoidal nerve (CN V_1_) Ex, exoccipital f, footplate of stapes fc, fenestra cochleae fi, fossa incudis fm, fibrous membrane of the tympanic cavity fn, facial nerve (CN VII) Fr, frontal fs, facial sulcus fsb, fissura basipterygoidea fv, fenestra vestibuli g, gonial gg, geniculate ganglion (CN VII) gpn, greater petrosal nerve (CN VII) H, hyoid hy, hypophysis iam, internal acoustic meatus ica, internal carotid a. icn, internal carotid n. icv, internal carotid v. ijv, internal jugular v. In, incus (= anvil) inf.a.a., inferior alveolar a. inf.a.n., inferior alveolar n. (CN V_3_) inf.a. v., inferior alveolar v. Ipa, interparietal ips, inferior petrosal sinus lama, lamina alaris lamb, lamina basalis lamh, lamina hypophyseos lamp, lamina parietalis lhv, lateral head v. (= vena capitis lateralis) ling.a., lingual artery long.c.m., longus capitis m. lp, lenticular process of incus lsc, lateral semicircular canal lt, lamina trabecularis lta, lamina transversalis anterior m., muscle Ma, malleus (= hammer) man, manubrium of malleus mas.m., masseter m. mbl, membrane limitans Mc, Meckel’s cartilage mdc, mandibular canal med.pt.m., medial pterygoid m. mso, membrana sphenoobturatoria Mx, maxilla mxa, maxillary a. mxv, maxillary v. mxt, maxiloturbinate my.m., mylohyoid m. n., nerve Na, nasal nc, nasal cavity nld, nasolacrimal duct npd, nasopalatine duct nph, nasopharynx ns, nasal septum ob, outer bar oc, oral cavity oph, oropharynx ot, otic capsule Pa, parietal paa, element of Paaw par, paraflocculus of cerebellum pars cochl, pars cochlearis Pe, petrosal pga, postglenoid artery pgv, postglenoid vein ph.con.m., pharyngeal constrictor m. ph.n., pharyngeal nerve ph.v., pharyngeal vein pm, tissues of the membranous meatus pn, paries nasi ppc, paraseptal cartilage ppo, pila praeoptica pr, promontorium pr.a, processus alaris pr.ep, processus epitympanicus pr.la.v, processus lateralis ventralis pr.m, processus medialis (= medial process of Sq.) pr.ma, processus mastoideus prs, prootic sinus psc, posterior semicircular canal Pt, pterygoid Px, premaxilla Rc, Reichert’s cartilage rl, recessus lateralis rtpp, rostral tympanic process of petrosal s, stapedius m. sa, sacculus saf, subarcuate fossa sev, sphenoparietal emissary vein Sq, squamosal ssc, septum spirale cartilagineum St, stapes (= stirrup) stclm.m., sternocleidomastoideus m. stm, secondary tympanic membrane sty, scala tympani of the cochlea sty.m., stylopharyngeous m. sve, scala vestibuli of the cochlea te.m., temporalis m. tg, trigeminal ganglion (= Gasserian or semilunar ganglion, CN V) tgty, tegmen tympani tm, tympanic membrane (= eardrum) tn, tectum nasi ton, tongue tt.m., tensor tympani m. (or its tendon) tt.n., tensor tympani nerve (CN V_3_) tvp.m., tensor veli palatine m. ty.n., tympanic nerve (CN IX) utr, utriculus v., vein vf, vestibular fossa VNO, vomeronasal organ (= Jacobson’s organ) Vo, vomer z, zygomatic process Other abbreviations L, left R, right CRL, crown-rump length HL, head length PND, postnatal day TL, total length	

## Chondrocranium: general features

De Beer ([1]:p. 465) summarized much of the previous work on the mammalian chondrocranium and presented a list of marsupial features compared to those of “reptiles”, monotremes, placentals, as well as ones unique to them (Table 2). This was the first comprehensive summary of the chondocranial anatomy of mammals, later followed by Roux [70] and Starck [71], whose contributions were also broadly comparative. Despite certain limitations, de Beer’s [1] work on mammals is an excellent starting point. His statements form the basis of a research programme. Many of the listed features are discussed in the present paper. Below, we elaborate on another summary, Table 3, regarding hypotheses about the ethmoidal region [94].

**Table 2:** Features listed by de Beer [1] as characteristic of marsupials. Several of the features do not concern the chondrocranium directly, but are listed here for the sake of completeness. The titles of each of three sections are taken from de Beer’s [1] formulation

“Reptilian” features of marsupials that are shared with monotremes and are absent in placentals ([1]:465): “the basal plate is broad” “the cochlear capsules are small” “the canalicular capsules lie directly above the cochlear, and the medial walls of the auditory capsules thus form the main part of the lateral walls of the cranial cavity in the posterior region” “the parietal plates are low” “the occipital arches are vertical” “the tegmen tympani is very small and there is no lateral prefacial commisure” “the incus lies dorsally to the malleus” “the stapes is columelliform” “the orbital cartilage and the sphenoethmoid and orbitoparietal commisures form a wide band” “the carotid foramina pierce the trabecular plate; i.e. no alicochlear commissures” “the internal carotid arteries enter the cranial cavity directly” “no mesethmoid bone is developed (feature shared by certain placentals)” “no ethmoidal cells or sinuses are excavated (except in *Phascolarctos*)” “the premaxillae bear a dentinal egg-tooth…”
“The characteristic features of the developing marsupial skull apart from those shared with monotremes (see above) and with placentals (see below)…are not clear cut” ([1]:466): “The presence of two pairs of hypoglossal foramina (feature shared with some rodents and Sirenia)” “The presence of a foramen rotundum between the ala temporalis and processus ascendens (feature not present in *Didelphis*, *Dasyurus*; shared with some Carnivora and Primates)” “The loss of the pila metoptica” “The diagonal position of the plane of the foramen olfactorium” “The small size of the frontals” “The large size of the lachrymals” “The great length of the jugals” “The poor development of the palatine process of the maxillae” “The extension of the alisphenoid to form a bulla surrounding the tympanic cavity” “The inflection of the posterior angle of the dentary” “The outer bar to Jacobson’s capsule (shared by *Dasypus*, *Tupaia*, *Macroscelides*, *Chrysochloris*, *Orycteropus*)” “The papillary cartilage (shared with *Tupaia*, *Macroscelides*, *Miniopterus* and some Rodentia)”
“… features shared by marsupials and placentals…” ([1]:466): “The subdivision of the foramen olfactorium advehens to form the cribiform plate” “The substitution of the fenestra rotunda and aqueductus cochleae for the foramen perilymphaticum” “The presence of a crista semicircularis in the nasal capsule” “The loss of the pila antotica” “The loss of the septomaxilla (except in the Xenarthra)” “The fusion of the reptilian pterygoid (secondary pterygoid cartilage) with the lateral wing of the parasphenoid” “The fusion of the cartilaginous elements of the 2^nd^ and 3^rd^ branchial arches to form thyroid cartilages”

**Table 3:** Features of the ethmoidal region hypothesized to be part of the marsupial “Grundplan” by Freyer [94], based on her critical assessment of ontogenetic and comparative anatomical data of the group

A rostrally wide closed cupula nasi anterior A short processus cupularis The processus laterales ventrales continuously connected to the lower edge of the septum and a caudal transition into the lamina transversalis anterior A processus alaris superior supporting the sulcus alaris with its dorsolateral lamella Lack of a commissura alicupularis - no connection between the processus cupularis and the processus alaris superior An inferior septal ride A superior septal ride A spina mesethmoidalis Lack of a fenestra internasalis (unfenestrated septum nasi) A cartilago papillae palatinae A rostral zona anularis An incisura nasopalatina A rostral process starting from the medial coiling of the lamina transversalis anterior, which corresponds to the dorsal lamella of the cartilago paraseptalis Location of the lamina transversalis anterior on the same horizontal plane as the lower edge of the septum nasi (a “keel” is absent) A ridge process (sensu [49]), a process of the lamina transversalis anterior A fibula reuniens, dorsally framing the opening of the Jacobson’s organ The cartilago paraseptalis not closed to form a tube A fissura septoparaseptalis running along the whole extent of the cartilago parasetalis A medial separation of the cartilago paraseptales The Jacobson’s organ opening into the nasal opening section of the ductus nasopalatinus, there is a communicative connection between the Jacobson’s organ, the ductus nasopalatinus and the cavum nasi Opening of the Jacobson’s organ lies at its rostral pole (lack of a process rostral of the opening) A lamina transversalis posterior A caudal zona anularis A caudally closed cupula nasi posterior A marginoturbinale An atrioturbinale A processus posterior atrioturbinalis An incisura maxillo-atrioturbunalis A maxilloturbinale A processus anterior maxilloturbinalis A lamellar nasoturbinale A latero-medial glandular ridge is present, which stays separated from the superior septal ridge Four ethmoturbinalia An interturbinale between the 2. and 3. ethmoturbinale Three frontoturbinalia The processus uncinatus is connected to the paries and does not display a free process caudally A wide commisura orbitonasalis has formed, which is connected to the paries	

In general, the marsupial chondrocranium has been characterized as having a lamina basalis of great breadth, a relatively large size of the otic capsule, and absence of the pila metoptica ([1]; Figs. 17 and 18). The latter is well established, the relative sizes of the parts are apparent when comparing three-dimensional models of mammals, but proper quantitative comparisons have never been made. The elements that contribute to the central stem or basicranial axis of the chondrocranium include the lamina trabecularis (=pars trabecularis), the lamina hypophyseos (=hypophyseal plate) representing the Anlage of the basisphenoid, and the lamina basalis (=basal plate) representing the Anlage of the basioccipital [70]. The boundary between the pre-chordal and chordal domains [96], and also that between the neural crest and mesoderm, is located at the basisphenoid-basioccipital synchondrosis, as determined in a study on the laboratory mouse *Mus musculus* [97].

Several skull foramina are landmarks that help to identify some bones in the adult stage when sutures are obliterated. In most marsupials the internal carotid artery enters the skull through a carotid foramen located entirely within the basisphenoid, as in monotremes [30, 47, 65]. In some marsupials (e.g., *Macropus eugenii*), outgrowths of the basisphenoid/alisphenoid and wings of the pterygoid may secondarily enclose the artery; consequently the aperture is seen in ventral view immediately in front of the basisphenoid/basioccipital synchondrosis, as recorded in macerated skulls [65]. However, the carotid canal always crosses basisphenoid territory, as in other marsupials (Fig. 13). The foramen rotundum for the maxillary division of the trigeminal nerve (CN V_2_) opens in the alisphenoid [30, 63]. The transverse canal, for a vein that communicates with the cavernous sinus, lies entirely within the basisphenoid [30, 98]. The sphenorbital fissure, a large gap that transmits nerves and vessels from the cavum epiptericum, opens on the medial wall of the orbit, between the orbitosphenoid and alisphenoid [30]. The ethmoidal foramen for a branch of the ophthalmic artery and the ethmoidal nerve (a branch of the ophthalmic division of the trigeminal nerve —CN V_1_) [29, 30] is usually in the suture between the frontal and orbitosphenoid, as reported for *Didelphis albiventris*, *Dasyurus maculatus*, *Monodelphis domestica* and *M. brevicaudata* [30].

The oval foramen, the aperture that transmits the mandibular division of the trigeminal nerve (CN V_3_) from the middle cranial fossa to the outside of the skull, shows different patterns within marsupials [34, 99, 100]. The name foramen ovale is the primary exit of CN V_3_ usually through an opening between the alisphenoid and the petrosal bone, or through the alisphenoid bone [99].

Outgrowths of the alisphenoid tympanic process may secondarily enclose CN V_3_ [99, 100]. For this condition, an incomplete enclosure (i.e., presence of a secondary foramen, but not a canal) and complete enclosure (presence of a canal continuous with the primary aperture of CN V_3_) has been recorded in didelphids ([100]: 30). The plesiomorphic condition for metatherians is the exit of CN V_3_ between the alisphenoid and petrosal bones (e.g., *Mayulestes*, *Pucadelphys* and *Andinodelphys* [14, 101]). Outgrowths of the alisphenoid enclosing the CN V_3_ have been found in different taxonomic groups (e.g., Didelphidae, Dasyuridae, Thylacosmilidae and other Sparassodonta [100, 102, 103]).

## The nasal region

The general studies of the chondrocranium cited above included sections on the ethmoidal region [42]. Some of Broom’s [52, 104] contributions are devoted exclusively to this area. Currently, the complexity and disparity of the ethmoidal region among mammals is being investigated intensively using non-invasive imaging [105], including marsupials [35]. Rowe et al. [95] and Macrini [11] presented detailed studies of its ontogeny in the didelphids *Monodelphis domestica* and *Caluromys philander*, respectively. In her unpublished Master’s thesis, Freyer [94] studied histological developmental series of *M. domestica*, and included a summary of 43 characters on the development of the ethmoidal region based on observations and a critical assessment of the literature for marsupial taxa. Freyer presented a list of features hypothesized to be part of the marsupial “Grundplan” sensu Hennig [106], meaning a list of character states for the last common ancestor of Marsupialia that are reconstructed based on phylogenetic considerations, and that included both plesiomorphic and apomorphic states in the context of mammal phylogeny (Table 3).

**Fig. 19:**
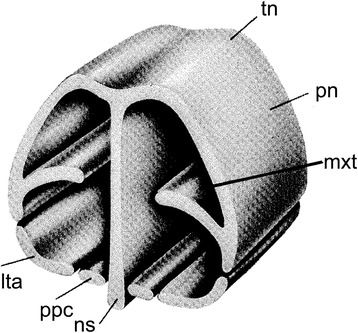
General schematic view of the anterior part of the nasal chondrocranium indicating its major features. Modified from Klima [216]

**Fig. 20:**
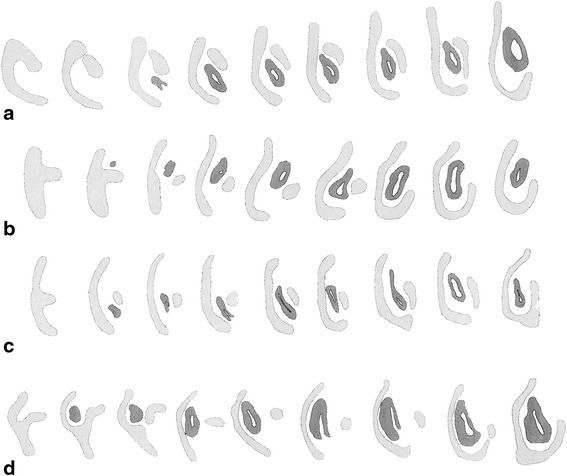
Schematic view of consecutive cross sections of the left paraseptal cartilage (*light*) and vomeronasal organ (*dark*) in **a** *Caluromys philander* (ZIUT-nn), **b** *Sminthopsis* sp. (ZIUT-nn), **c** *Thylogale billardieri* (ZIUT, HL 13 mm) and **d** *Dromiciops gliroides* (ZIUT, HL 19 mm). Not to scale. Notice that the relations of the *outer bar*, the vomeronasal organ and the main body of the paraseptal cartilage, are similar between the dasyurid (**b**) and *Dromiciops* (**d**). Australian marsupials and *Dromiciops* (**b**, **c**, **d**) have a dorsal extension of the paraseptal cartilage with regard to the *outer bar* that is lacking in didelphids (**a**)

The overall construction of the nasal floor in marsupials is uniform, and all species studied to date have shown (1) presence of a vomeronasal organ and nasopalatine duct, (2) absence of a nasopalatine duct cartilage, and (3) a palatine cartilage absent or incipient (Fig. 19). Several other features of the nasal floor are variable and homoplastic across species, including the presence of glandular ridges and of an isolated dorsal process of the paraseptal cartilage [48, 107], while others represent features of potential phylogenetic significance. Sánchez-Villagra [48] presented a study of structures around the vomeronasal organ, including a matrix of 16 characters across species representing 13 “families” and six “orders” of marsupials. Some of the main conclusions are summarized as follows. The opening of the VNO into the upper end of the nasopalatine duct was present in the marsupial Grundplan (Figs. 2 and 3). Most marsupials have a large and horizontal anterior transverse lamina, the plesiomorphic condition, which becomes oblique in diprotodontians. Among the autapomorphies of clades found are the conspicuous internasal communication of perameliformes, the “tube-like” or ring-shaped paraseptal cartilage of vombatiformes, and an “anterior upper chamber” in the nasal cavity of *Caenolestes* sp., a structure forming from the contact between the superior septal ridge and a more inferior glandular-rich extension of the turbinal region [94, 104]. A feature of *Caluromys philander* and of Australasian marsupials and *Dromiciops*, with the exclusion of perameliformes, is the middle and not dorsal connection of the outer bar to the paraseptal cartilage (Fig. 20).

**Fig. 21:**
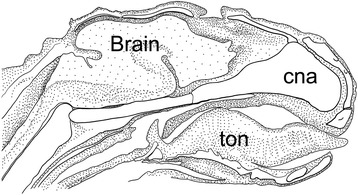
A schematic sagittal section serves to illustrate the whole of the head at postnatal age 1-day in the opossum *Monodelphis domestica* (drawing based on photograph published by Maier [75], p.62). The epiglottis reaches the dorsal side of the velum. Notice the lack of a chondocranial coverage of the dorsal brain, present in monotremes [12, 45]

A massive and broad cupula nasi anterior is characteristic of marsupials around the time of birth (Figs. 2 and 21) and can be hypothesized to be related to the perinatal biology of marsupials. Freyer [94] reported nerve bundles in the epithelium rostral to the cupula nasi anterior of *Monodelphis domestica*, possible nerves of CN V_2_ that innervate the area which later develops into the rhinarium [108]. Previous studies have hypothesized a causal relation between the innervation and sensory anatomy of the area proximal to the cupula nasi anterior and the sensory biology of marsupials at birth [109–114]. Taking eye development as a reference for comparing stages, Elsner [115] observed that the cupula nasi anterior develops much earlier in *Monodelphis domestica* than in the tree shrew *Tupaia belangeri*. The nasal openings in *Monodelphis* are laterally oriented at birth, which also makes functional sense.

A prominent structure in the nasal region of the mammalian chondrocranium is the nasal septum (Figs. 2 and 3), which is not fenestrated in marsupials, in contrast to the condition of some placentals [65, 116]. One of the main unresolved issues in mammalian cranial comparative anatomy concerns the ossifications at and around the nasal septum [11, 70, 117, 118]. The ossification of the nasal septum can spread from the presphenoid, as reported for many marsupial taxa by Broom ([52, 104], although in some cases incorrectly interpreted), or it can be a separate, more rostral ossification, as reported by Rowe et al. [95] for *Monodelphis domestica*. The studies that have attempted to distinguish these two options, and thus decide if a mesethmoid is present or not, as discussed by Ferigolo [119], can suffer from the biases introduced by the stages examined, which may prevent the recognition of the actual pattern. Starck ([18], p. 43) wrongly supposed the “mesethmoid = ethmoid”, inferring that ossification from a cartilaginous precursor in the posterior and dorsal portion of the nasal septum leads to this replacement bone.

The number of ossification centers are not necessarily reliable guides to homology in other areas of the skull, and examples of homologous bones with different numbers of ossification centers across species abound [1, 120]. In the case of the mesethmoid, there is a terminological agreement—a separate ossification from the orbitosphenoid is by definition the sign of the existence of a mesethmoid [52]. The mesethmoid joins caudally the orbitosphenoid at a point near the optic chiasm. Confusion of the mesethmoid with other bones in that area of the ethmoidal region is easily avoidable. The ethmoid is a paired bone, whereas the mesethmoid is unique and median in its position; the v-shaped and unpaired vomer has clearly a more ventral position. Rowe et al. [95] reported for *Monodelphis domestica* that, whereas the mesethmoid ossifies in the third postnatal week from a single, median endochondral center, the ethmoid is paired and grows via the coalescence of multiple bilateral perichondral ossifications. As these elements co-ossify in adults, their distinctiveness and the homologies across taxa have been a source of disagreement and contradictory assessments in the literature. In one detailed study of its ethmoidal region, the mesethmoid is reported as lacking in *Caluromys* [11].

Understanding the transformation along the synapsid line in the area in which the ethmoidal and orbital region became closely connected (e.g., [121–123]) could provide important clues on the homologies of the bones involved, in parallel to the developmental approach taken in this paper and to comparative anatomical comparisons of extant forms. The fossil record documents the merging of wings of the orbitosphenoid to become the presphenoid. The ethmoid may represent a unique case of a true neomorph in the mammalian skull (cf. [123]; unpublished). The presence of the parasphenoid is less known in marsupials, as it appears when present as a very small ossification ventral to the basisphenoid-basioccipital suture (e.g., [124, 125]) and as we have observed in a section of *Didelphis* sp.

The timing and number of centers of ossification of the bones of the orbitotemporal area adjacent to the ethmoidal region have been documented in *Monodelphis domestica* ([63], as summarized by [30]). The presphenoid (midline rod) and orbitosphenoids (arms) arise on postnatal days 13 and 14 from three ossification centers that fuse to form a T-shaped structure by postnatal day 16. The basisphenoid arises from a single center of ossification on postnatal day 5. Each alisphenoid arises from two centers of ossification on postnatal day 4, both fusing by postnatal day 7 (cf. [57]). The basisphenoid and alisphenoid are fused together by postnatal day 25 ([30], page 151).

## Primary braincase wall and other chondrocranial features

The sidewall of the primary braincase of tetrapods is formed by three vertical cartilaginous pillars: the pila praeoptica, pila metoptica and pila antotica, which during ontogeny grow between certain cranial nerves [1]. In placentals, the pila antotica is absent, leaving a common opening for the exit of CN III—CN VI. In marsupials, the pila metoptica and pila antotica are absent, which results in a large opening for CN II—CN VI (e.g., [12, 45, 73, 74, 126]). Although CN II is included within the sphenorbital fissure only in marsupials, a similar opening in placentals is called by the same name. In placentals, CN II is enclosed in a separate optic foramen [127].

In addition to the pilae, the other elements that contribute to the primary braincase wall in marsupials are the ala orbitalis, commissura orbitonasalis, commissura orbitoparietalis and commissura suprafacialis (Fig. 17). The braincase wall is completed by the secondary wall; the ala temporalis with the lamina ascendens (= ascending process) and the membrana sphenoobturatoria, the last two Anlagen of the alisphenoid [73, 74] (Fig. 16). Although the secondary wall is not part of the chondrocranium [128], it is relevant to discuss it here.

The cartilaginous ala temporalis of marsupials is homologous to the basal and ascending processes of the palatoquadrate of reptiles [42, 57]. The alisphenoid starts to grow intramembranously from the perichondrium of the ascending process of the ala temporalis, appositionally on the membrana sphenoobturatoria, and endochondrially within ala temporalis itself ([42, 57, 58]; Fig. 16).

The ascending process of the ala temporalis has different relationships with the branches of the trigeminal nerve (CN V) among marsupials (Table 4). The differences in the relationships between the ala temporalis and the branches of the trigeminal nerves in mammals triggered extensive discussion regarding the homology of the alisphenoid bone [76, 128]. Peripheral nerves develop much earlier than the first skeletal structures and even homologous cartilaginous and bony elements may develop topographically in different ways to accommodate the primary organization of the head organs [42, 57, 74].

**Table 4:** Relations of the ala temporalis to the branches of the trigeminal nerve in several marsupials. *Trichosurus* A: “young”. *Trichosurus* B: “older”

Taxon	Between V1 & V2	V2 traverses ala temporalis	Between V2 & V3	Source
Didelphidae				
*Monodelphis*	X			Maier, 1987 [57] Sánchez-Villagra 1998 [65]
*Didelphis*	X			Fuchs, 1915 [208] Presley, 1981 [78] Maier, 1987 [57]
*Philander*	X			Maier, 1987 [57]
*Caluromys*	X			Maier, 1987 [57]
Dasyuridae				
*Sminthopsis*		X		Maier, 1987 [57]
*Dasyurus*			X	Broom, 1909 [51] Maier, 1987 [57]
Peramelidae				
*Perameles*	?X			Maier, 1987 [57]
*Isoodon*		X		Esdaile, 1916 [56] Maier, 1987 [57]
Phalangeridae				
*Trichosurus* A		X		de Beer, 1926 [209] Goodrich, 1930 [210] Sánchez-Villagra 1998 [65]
*Trichosurus* B			X	Broom, 1909 [51] Maier, 1987 [57]
Petauridae				
*Petaurus*			X	This work
Macropodidae				
*Petrogale*			X	Maier, 1987 [57] Sánchez-Villagra 1998 [65]
*Wallabia*			X	Klutzny, 1994 [60]
Acrobatidae				
*Acrobates*			X	Maier, 1987 [57]
Vombatidae				
*Vombatus*		X		Klutzny, 1994 [60] Sánchez-Villagra 1998 [65]

In many adult marsupials, the CN V_2_ exits the skull through its own aperture, the foramen rotundum. This can be a consequence of the ala temporalis eventually surrounding CN V_2_, to be replaced later by intramembranous growth of the alisphenoid (e.g., *Vombatus*, *Trichosurus*, *Sminthopsis*) or by appositional deposition of bone (e.g., *Didelphis*, *Monodelphis*, as in some placentals) [57, 74, 126, 128, 129]. The latter condition seems to be secondary, and the presence of a foramen rotundum may be a convergent feature of different groups of mammals [74].

The commissura orbitoparietalis is a bar of cartilage on the side wall of the cranium (Figs. 16 and 17). There is no clear boundary between it and the lamina parietalis. The lamina parietalis is a thick plate of cartilage; its ventral limit is the dorsal margin of the canalicular part of the otic capsule [54] (Figs. 5, 12 and 15).

The ascending process of the ala temporalis reaches the commissura orbitoparietalis early in ontogeny, forming part of the secondary lateral wall of the braincase (Fig. 16) and providing mechanical support to withstand the pull of the adductor muscles during suckling [42, 57]. The strong commissura orbitoparietalis and the membrana sphenoobturatoria provide initial attachment area for the strong temporalis muscle [57].

Early in ontogeny, the trigeminal ganglion is situated in the cavum epiptericum, between the membrane limitans and the membrana sphenoobturatoria [57, 60]. In neonates, the trigeminal ganglion is large, occupying most of the cavum epiptericum ([57]; Fig. 16). This precocious development of the trigeminal system might be interpreted as being related functionally to the suckling activity of the neonate [42, 57], as in the case of the buccinator muscle, which is innervated by sensory branchs of CN V_3_ and directly involved in suckling. However, monotremes also have a large trigeminal ganglion early in ontogeny, and they do not suckle [130].

The processus alaris is the lateral projection of the hypophyseal plate of the central stem [8] (Figs. 4, 11, 13 and 16) which becomes continuous with the ala temporalis [1]. In earlier ontogenetic stages, there is gap between the procesus alaris and the ala temporalis, the fissura basipterygoidea [42, 57, 58] (Fig. 16). In placentals, the processus alaris is often connected to the otic capsule by a cartilaginous bridge, the alicochlear commissure. The alicochlear commissure is characteristically missing in marsupials according to De Beer ([43; see also [57]).

In the posterior portion of the chondrocranium, the principal element is the pila occipitalis. This fuses with the lamina parietalis to form the portion of the chondrocranium which surrounds the foramen magnum. The occipital condyles are formed by the pila occipitalis. In the back of the chondrocranium, all marsupials studied to date are characterized by double hypoglossal foramina, as opposed to the single foramen characteristic of most placentals (Table 2; [65]).

## Lower jaw and middle ear ossicles

The transformations of the middle ear and associated jaw joint anatomy [131] are classic subjects in mammalian anatomy and paleontology [132–137], and conditions in marsupials have figured prominently in their interpretations [59, 138, 139]. The evolutionary transformation involves complex changes in the craniomandibular hinge from a primary quadrate-articular to a secondary dentary-squamosal jaw joint, and the progressive reduction and detachment of the postdentary bones as a series of ossicles transmitting the sound waves in the middle ear ([59, 140]).

Recently, Ramírez-Chaves et al. [141] reviewed and quantified several structures involved in this evolutionary transformation based on ontogenetic material for six species representing three major marsupial clades. The plesiomorphic mammalian pattern for the Anlagen of the middle ear bones is for them to be medially shifted from the mandible [42, 72]. In marsupials, this relocation is reportedly much less marked, which is considered derived [42, 141].

There is a displacement of the middle ear bones in the posterior direction during ontogeny in all mammals. Based on a study of *Monodelphis domestica*, it was suggested that this is associated with braincase enlargement and tied to the detachment of structures building part of the middle ear in the adult [21]. This hypothesis stimulated productive examination of rates of development in structures in different marsupials [142] and comparisons with the fossil record [143, 144]. Alternatively, Ramírez-Chaves et al. [141] presented evidence that dental function, as indicated by molariform tooth eruption, better correlates with the detachment and ossification of the middle ear ossicles than brain expansion, and that the negative allometry of the ossicles begins after their detachment.

**Fig. 22:**
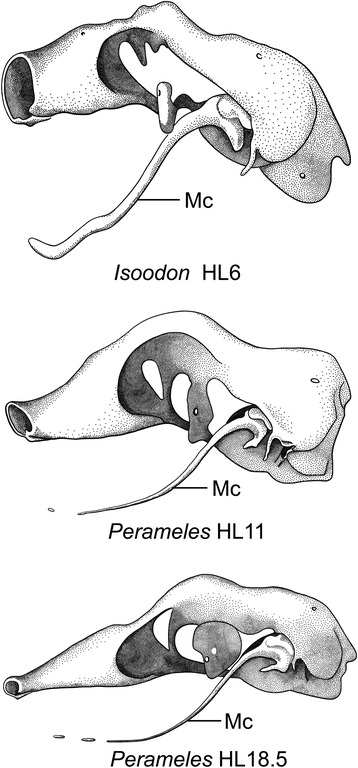
*Left side* of chondrocranium, without membrane bones, in bandicoots (Peramelemorphia) at different stages of development. Cardboard reconstructions, modified from Esdaile [56]. *Top*, cf. *Isoodon obesulus*, *Perameles obesula* in the original paper, Stage II. TL 15.5 mm. HL 6.0 mm (× 25.); *middle*, *Perameles nasuta*, Stage IV. TL 23.0 mm. HL 11.0 mm (× 12.); *bottom*, *Perameles nasuta*, Stage V. TL 35.0 mm. HL 18.5 mm (× 7.)

Meckel’s cartilage forms in early ontogenetic stages; in marsupials it is usually a conspicuous structure, reportedly relatively larger in relation to the rest of the developing skull than in placentals [145, 146]. This question deserves further examination. The size of the Meckel’s cartilage changes through development (Fig. 22), and some illustrated stages of placentals (e.g., *Rousettus aegypticus*, [18]) show a fairly robust Meckel’s cartilage. In early ontogenetic stages, Meckel’s cartilage is lodged in a longitudinal groove, the medial trough of the dentary, which becomes shallower or disappears in later ontogenetic stages [42]. In marsupials at birth, the lower jaw is still suspended by the primary joint, with the incus supported by Meckel’s cartilage [42, 147]. The incus is mobile relative to the cranium and functions as a jaw joint until the resorption of Meckel’s cartilage [132]. The resorption of Meckel’s cartilage [148] occurs postnatally and results in the definitive separation and differentiation of the malleus [141] (Fig. 22).

Ramírez-Chaves et al. [141] found that “middle ear detachment” (i.e., the disappearance of Meckel’s groove) is allometric, occurring earlier in smaller species and later in larger ones. The relation of the middle ear to the size of the mandible during ontogeny has been generally assumed to be negatively allometric [59]. Ramírez-Chaves et al. [141] found that this kind of proportion exists only after ossification of the ossicles has been nearly completed.

## Tegmen tympani

The tegmen tympani is a process or “wing”, initially formed in cartilage, that originates in continuity with the anterolateral part of the pars canalicularis of the auditory capsule [8, 149]. Originally named by Hyrtl [150], it forms part of the caudal roof of the tympanic cavity, where it is crossed by the facial sulcus [1, 8].

The tegmen tympani has been regarded as a neomorphic feature of therians [25, 74]. Rougier et al. [151] hypothesized that this feature is present in *Vincelestes*, a stem therian from the Early Cretaceous of Argentina. In marsupials, the tegmen tympani is a rudimentary structure and is somewhat inconspicuous in the adult (e.g., [1, 41, 45, 53, 55, 66, 126]). According to Van Kampen’s ([25]:345) comment on the subject, in marsupials and bats the *anterior part* of the tegmen tympani is highly variable and often missing (“Der vordere Teil des Tegmen tympani ist sehr veränderlich und fehlt nicht selten ganz”). This has been misinterpreted as meaning that the entire structure is absent in marsupials. Clearly, however, Van Kampen [25] described its presence and condition in several marsupials, including didelphids ([25]:398; regarding *Didelphis marsupialis*, see Toeplitz [53] and de Beer [1]:301, but see also Aplin [66]), peramelids ([25]:401), phascolarctids ([25]:407), and macropodids ([25]:414). More recently, an incipient tegmen tympani has also been noted for the young of *Thylogale* [42], *Phalanger* [152], *Cercartetus* [66] and *Macropus* [62, 66]. We report here a small tegmen tympani restricted to the rear of the roof of the epitympanic recess in *Macropus eugenii* (Figs. 13d and 14a). In the specimen studied, the ossification process has started on the lateral part of the commissura suprafacialis and the adjacent part of the pars canalicularis. The tegmen tympani is continuous with both of these structures. Schmelzle [62] (page 49, fig. 51), named this ossifying area in *Macropus eugenii* the “commissura suprafacialis lateralis”, formed by replacement (one with a cartilaginous precursor) and appositional bone (“Zuwachsknöcherung”), but for clarity it is important to note that the commissura suprafacialis lateralis is structurally continuous with the tegmen tympani. Additionally, the ridge that he called the “processus epitympanicus cochleae” ([62]:22) appears to merely be the lateral edge of the commissura suprafacialis and does not require a separate name. In addition, Schmelzle [62] recorded differences in the ossification mode of the tegmen tympani in various species, but these do not affect the homology of the structures concerned.

Aplin [66] discussed the tegmen tympani critically with new information on several species. Aplin ([66]:6–37) reported that in pouch young of *Macropus* and *Cercartetus* “a fibrous, fascial membrane”, which we identified as the membrana epitympanica (see below), roofs over the auditory ossicles. He noted that the membrane contains a “nodular piece of cartilage” maturing later than the surrounding skeletal structures and which he identified as the tegmen tympani. However, this is unlikely since the tegmen tympani has never been seen to originate as an independent element in other mammals. It may be that the nodular piece of cartilage is related to structures occasionally seen in many mammals in this area of the developing skull of (e.g., the element of Paaw: the tendon of the stapedius muscle, Fig. 15a; the element of Spence: a skeletal element associated with the chorda tympani; [8]). In this context also, for *Trichosurus vulpecula* Broom [51] named a cartilaginous ridge projecting downwards from the otic capsule and apparently separated from it, which he identified as the tegmen tympani. As illustrated ([51]:fig. 22), this interpretation cannot be correct. The ridge is either Reichert’s cartilage (second pharyngeal arch) or, as Broom himself noted ([51]:201), possibly a projecting part of the exoccipital.

In the macerated skull of *Monodelphis brevicaudata*, the tegmen tympani is seen as a small process, lateral to the facial nerve. This structure has sometimes been identified as the tuberculum tympani, but it is the precise equivalent of the tegmen tympani of placentals ([30]:160, fig. 7).

It seems that for marsupials detailed studies of fine-grained ontogenetic series are necessary to establish the nature of the small processes and structures in the area identified as tegmen tympani. Although such detailed comparative anatomical research is relevant, it is unlikely to provide significant results in the form of well constrained systematic characters. At microscopic scale, there is proved to be much diversity in the extent of structures and their mode of ossification. Trying to incorporate such details into character definition for phylogenetic analysis is likely to be unfruitful [153].

## Tympanic cavity and tympanic floor soft tissues and bony elements

The cavum tympani is a mucous-membrane lined space that fills the osseous middle ear cavity and into which the auditory ossicles project [8]. During ontogeny, the cavum tympani expands. As it does, the mucoid tissue filling the cavity is resorbed until the cavum attains the middle ear limits. In the embryo, these limits are defined by membranes; the membrana epitympanica at the roof, the fibrous membrane at the floor, and the tympanic membrane (=eardrum). The definitive tympanic cavity (=middle ear cavity) is the osseous chamber containing the cavum tympani [8]. The tympanic cavity can also include the pneumatization from the surrounding basicranial elements [73].

The proliferation of cells within the mesenchyme surrounding the expanding cavum tympani forms the fibrous membrane of the tympanic cavity [8], which is continuous with other connective tissue lining the basicranium (including the tissues of the membranous meatus in Figs. 6e, f, 11f and 13b). Cartilaginous or bony auditory bulla elements will later develop within this membrane or adjacent to it [74]. This tympanic floor also separates the tympanic space and contents from surrounding structure. In the adult it can be membranous, or consist of cartilaginous and/or bony structures. In adult marsupials, as many as three bones can form the tympanic floor; the alisphenoid (alisphenoid tympanic process), the ectotympanic and the petrosal (rostral and caudal tympanic processes petrosal; RTPP and CTPP, respectively) (e.g., [78, 98, 154–158]). The possible presence of an entotympanic, as a fourth bone in the tympanic floor, is discussed below.

A membranous bulla forming the tympanic floor is interpreted as the plesiomorphic condition for marsupials, as no bony floor is present in most Mesozoic and Cenozoic stem metatherian species (e.g., [29, 101, 103, 159–161]). There are however some exceptions (e.g., *Asiatherium* and herpetotheriids, probably the sister-group of crown marsupials [162]) that did have an alisphenoid bulla [162, 163]. Although classically considered to be a marsupial autapomorphy (e.g., [73, 157, 164]), the presence of an alisphenoid contribution to the floor of the tympanic cavity is a derived state that occurred independently in different metatherian lineages [14, 34, 103, 158, 162, 163]. As is usually the case with tympanic floor components other than the ectotympanic, the formation of the alisphenoid tympanic process appears to be a late event (e.g., in *Monodelphis domestica* it happens around PND-35, only 15–20 days before weaning [165]).

Bullar ontogeny has been documented for several marsupials (e.g., *Monodelphis domestica*, *Didelphis marsupialis*, *Caluromys philander*, *Perameles nasuta* and *Isoodon obesulus*, *Dasyurus viverrinus*, *Vombatus ursinus* and *Macropus eugenii* [9, 41, 53–57, 60, 62, 66]). Additional information from macerated skulls, presented in an ontogenetic perspective by Maier [31] has been gathered for six didelphids, one dasyurid and two diprotodontians.

The entotympanics represent an “assortment of non-homologous entities” [166–168], that are “bony or cartilagenous, that lie in the ventral wall of the tympanic cavity and are ontogenetically primarily independent of the other elements in the auditory bulla, except perhaps the tympanohyal and the cartilage of the Eustachian tube [= tubal cartilage]” [8, 26, 166, 168–174]. Following van der Klaauw [26, 169], two types of entotympanics are distinguished based on their approximate site of formation: rostral and caudal. The rostral entotympanic most commonly develops in close relation to the tubal cartilage and is primordially continuous with it (e.g., in the vespertilionid bat *Miniopterus*, as described by Fawcett [175]; *Elephantulus fuscipes*, [8]). The caudal entotympanic appears in the rear part of the tympanic floor, sometimes but not always in relation to Reichert’s cartilage. It extends between the ectotympanic and the petrosal.

Entotympanics have figured prominently in many discussions of mammalian systematics [168]. Many authors have pointed out that entotympanics have no evident homologues among non-eutherian mammals or other tetrapods and are placental neomorphs ([27]; [166]:38; [17, 168]). However, numerous reports of entotympanics in marsupials exist in the literature (see Maier [31] for a review). Parker ([69] cited in Winge [27]:122) noted that many marsupials have what he called “Os bullae” (=entotympanics). Carlsson [176] and Wood-Jones [177] reported an entotympanic in the dasyurid *Dasycercus* spp., as Van der Klaauw [26] did for *Perameles* sp. and *Vombatus* sp., and Segall [154–156, 178]) reported its occurrence in *Didelphis* sp., *Caluromys* sp., *Dactylopsila* sp., *Isoodon obesulus* and (as in Hershkovitz [179]) *Dromiciops gliroides*. The presence of a septum inside the bullar wall (“septum sphenoideum”) in a number of phalangeroid genera was cited by Segall [178] as the division between the tympanic process of the alisphenoid and the entotympanic. But none of these reports of entotympanics in marsupials have been substantiated with detailed descriptions of adult skulls or developmental data. Archer [29] did not find entotympanics in his exhaustive review of the (adult) marsupial basicranium.

**Fig. 23:**
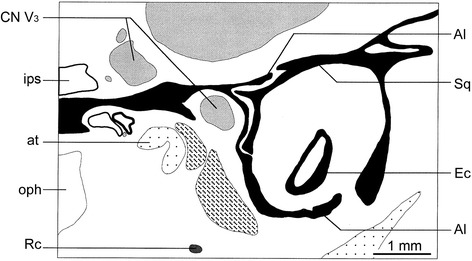
Schematic drawing of the cross section of the basicranium of *Dactylopsila* sp. (DUCEC-8323, section 1313 Plate 6a). This species exhibits the typical bullar condition of most diprotodontians, consisting of a squamosal roof of the middle ear, and a squamosal process contributing to the lateral side of the bulla overlain medially in part by a tympanic process of the alisphenoid

Examination of dozens of macerated skulls of *Didelphis* spp. revealed that what Segall [178] called an entotympanic in this species is actually the RTPP, the pars petrosa of Patterson ([180]) [65]. Also, there is no conclusive evidence of an entotympanic in *Dactylopsila* sp. (DUCEC-8323), contra Segall ([156]: 197; [178]: 27, 41, 42). *Dactylopsila* sp. has the typical bullar condition of most diprotodontians, consisting of a squamosal roof of the middle ear, and a squamosal process contributing to the lateral side of the bulla overlain medially in part by a tympanic process of the alisphenoid (DUCEC-8323, Fig. 23). This condition was labeled as “partially bilaminar” by Murray et al. [32] and can be discerned only by looking at sectioned material. In the extinct vombatiform *Wakaleo vanderleuri* this condition becomes more accentuated in that the squamosal fully surrounds the middle ear cavity [32]. This greater contribution of the squamosal characterizes wombats, which in addition show almost no remnant of an alisphenoid tympanic process [28].

Presley [181] described an “entotympanic-like” element in *Trichosurus vulpecula*, which he attributed to an early contact between Reichert’s cartilage and the auditory capsule, but he was referring actually to the RTPP [8, 67]. Presley [182] also described this contact between Reichert’s cartilage and the cochlear capsule in *Didelphis*, but Maier ([31, 41]) found no evidence of such contact in the close relative *Monodelphis* and in other marsupials. In all cases, the RTPP was formed by periosteal outgrowth from the ossifying cochlear capsule (Maier [31, 41]), as described for the RTPP in a variety of placentals [8]. The only two well-substantiated reports of entotympanics in marsupials deserve discussion.

Norris [183] studied macerated skulls of different ages of *Phalanger orientalis* and found that the bulla remained membranous even at the stage when the third molar erupted. He recorded three areas of incipient ossification; the first two are in the alisphenoid. The third and caudalmost ossification is described as an element within the bullar membrane independent of any of the surrounding bones; it is therefore (according to Norris) an entotympanic. Norris [183] reported that by the time the fourth molar erupts, the rapid ossification of these centers obliterated any sign of independent ossifications in the floor of the bulla.

Aplin ([66]:5–25) mantained, on the basis of a study of macerated ear regions of *Acrobates* and cranial sections of a pouch-young *Distoechurus*, that the anterior bullar element of these species is an intramembraneous ossification “quite distinct from the cartilage of the auditory tube”, which he referred to as an entotympanic. As described by Aplin [66], this element corresponds to the rostral entotympanic of some placentals. However, the typical placental rostral entotympanic forms initially in cartilage and chondrifies in direct continuity with the tubal element (some exceptions in Wible and Martin [184]), whereas the nature of the element in acrobatids seems quite distinct. Such differences aside, the anterior bullar element of acrobatids might represent the first (and only) independent intramembraneous entotympanics-like element to be reported in any marsupial [66].

The basicranium of *Dromiciops* is of particular interest. It had been studied by Segall [154] through examination of macerated skulls of adults. We examined histological sections of a juvenile of CRL = 37 mm (ZIUT, HL 19 mm) (Figs. 6, 7, 8, 9 and 10), see also Sánchez-Villagra and Wible [98]. In spite of the fairly advanced state of ossification of this specimen, the high resolution of the sectioned material allows a rejection of the specific interpretations of Segall [154] and Hershkovitz [179] about the possibility of an entotympanic in *Dromiciops* (lack of an entotympanic was reported but not documented by Maier [31]). This conclusion is reached in conjunction with observations of dozens of macerated skulls of adults at the Field Museum of Natural History [65]. As identified by Sánchez-Villagra and Wible ([98], fig. 11) in a macerated skull, the auditory bulla of *Dromiciops* is clearly formed by three components: (1) the anteriormost element is the tympanic process of the alisphenoid (Fig. 6), (2) the middle element is the RTPP (Fig. 8), and (3) the posterior element is the CTPP, with a medial prong projecting anteriorly.

In *Dromiciops*, the ectotympanic forms a nearly complete ring inside the bulla (in an aphaneric [not visible from the external surface] position), and does not contribute to the bulla in ventral view. As described by Hershkovitz [179]) and also recorded here, the basioccipital expands laterally to overlap (partially cover) the medial side of the bulla. Accordingly, two layers of bone are present in this area: the medial bullar compartment and the basioccipital ventral to it. Even though they did not provide developmental data to support their claims, other authors have correctly assessed the bullar composition in *Dromiciops*. Reig and Simpson ([185]:525) named the medial compartment as “pars petrosa”, while Szalay ([17]:75) called it the “petrosal wing” (= RTPP of this work; Fig. 8b–d). The posterior compartment was named “pars mastoidea” (= CTPP; Fig. 10b) by Reig and Simpson ([185]:525) as well as Patterson [180].

A similar condition to that of *Dromiciops* with respect to bullar composition is found in the honey possum *Tarsipes*. The latter exhibits a complete tympanic floor formed by an alisphenoid tympanic process, and conjoined RTPP and CTPP ([66]:6–30).

In summary, no conclusive evidence of entotympanic bones was found in the tympanic floor of the marsupials examined. So far, this element has been considered a derived feature of placentals [165, 167]. The report of an entotympanic-like element in acrobatids [66] deserves further examination. Additionally, if an “entotympanic” element is present in the bulla of some *Phalanger orientalis* [183], this would add to the striking variability of bullar makeup in this species. We describe here the tubal element of *Dromiciops* (Fig. 7), because its histological nature resembles that of some placental taxa in which the rostral entotympanic forms in continuity with the tubal cartilage (e.g., *Elephantulus* [8]).

## Auditory tube and tubal element

The tubotympanic recess, a derivative of the first pharyngeal pouch [186], differentiates to become the tympanic cavity of the middle ear and the auditory tube. The auditory tube (=Eustachian or pharyngotympanic tube) is the channel of communication between the tympanic cavity and the nasopharynx that mediates pressure within the middle ear. Surrounding the auditory tube in some species is a differentiated cartilaginous auditory tube (CAT) or “tubal element”, most frequently described for placentals (e.g., [8]) and in the echidna, but not in the platypus [9, 187, 188]. Reports of cartilage in marsupials are contradictory [65].

In relation to the nature of the tubal element in marsupials, Wible ([9]:298) stated: “a cartilage of the auditory tube reaches medially from the anterolateral corner of the promontorium’s anterior pole and shields the rostral surface of the auditory tube in its passage to the pharynx in the developmentally older pouch young - *Perameles nasuta* (HL-H Ms235) and *Macropus rufogriseus* [his *“Wallabia rufogrisea”*] (AIF 7895) (figs. XI-7, XI-9). This element does not, at least in the forms investigated, contribute to the tympanic roof. To my knowledge, this is the first account of the cartilage of the auditory tube in marsupials”. This observation by Wible ([9]; see also Weber [188] and Edgeworth [189]) contrasts with the report by Maier et al. ([187]: 16) that “the auditory tube of didelphids (and possibly of all marsupials) is not framed by tubal cartilages, but only by dense connective tissue”.

Sucheston and Cannon [190] reported a fibrous/collagenous tubal element without hyaline cartilaginous tissue in an adult opossum *Didelphis marsupialis*, as part of an extensive histological study including eight placental species, for which they described several kinds of cartilage in the tubal element. Similarly, Aplin [66] reported a fibrous/collagenous tubal element in all but one diprotodontian marsupial of his extensive histological sampling of this group. In addition, Aplin [66] recorded in an adult of the honey possum *Tarsipes* a large tubal element containing chondrocytes, with the element serving as a major source of origin of the medial pterygoid muscle.

In terms of the specimens examined in this work, we report that the tubal element in most marsupials consists of mesenchymal tissue that resembles precartilaginous tissue, but no cartilage per se was seen (Figs. 4b, 11c, d and 13b). A reexamination of the specimen of *Macropus rufogriseus* that was described by Wible [9] as having a tubal cartilage may be in fact consist of dense connective tissue characterized by the proliferation of cell nuclei, but lacking chondrocytes or cartilaginous matrix ZIUT-nn, [65]: section 41-2-2; Figure IV-2a). In this case, an examination of developmental series that include relatively late stages will be needed to best characterize the tissues in question.

In *Dromiciops* the tubal element is definitely best described as a type of cartilage with high fiber content (Fig. 7). Whether it corresponds to the kind of fibrocartilage said to form the cartilage of the auditory tube in humans would have to be determined histochemically. As Ross et al. ([191]: 135) noted, “it is often difficult to distinguish fibrocartilage from dense regular connective tissue, particularly in hematoxylin and eosin stained sections”. Fibrocartilage is typically in places where tendons attach to bones, indicating that “resistence to both compression and shear forces is required of the tissue” ([191]:136; see Kummer [192]). In the specimens examined, including *Dromiciops*, the tensor veli palatini muscle is attached to the tubal element (Fig. 7a), as was reported by Aplin [66] for many diprotodontians and as is the case in humans [193]. Some of the histological characteristics of the marsupial tubal element are reminiscent of fibrocartilage, perhaps because of the mechanical requirements of that tissue, though it is only a matter of speculation what forces are produced by the tensor veli palatini muscle.

The appearance of the tubal element occurs late in placental ontogeny relative to overall chondocranial development. Proctor [194] reported that in humans the cartilage forming the tubal element appears during the fourth month of fetal life. Many perinatal specimens of marsupials show dense connective tissue in the shape of what would be the CAT in placentals, but this tissue has not seen to differentiate any further in marsupials.

The lack of any kind of cartilagenous element in didelphids and most other marsupials with the exception of *Dromiciops* and *Tarsipes* cannot be interpreted with certainty as primitive or derived. The cartilage of placentals might be homologous to an ancestral mammalian condition if present in the echidna and secondarily lost in the platypus. If the ancestral therian had a cartilaginous tubal element, then the lack of cartilage in marsupials may be the result of truncation in the process of cartilage formation.

## Conclusions and looking ahead

The study of histological sections is very time consuming and only the collections of extensive series is likely to deliver information that can serve to diagnose groups of organisms. This kind of work has been accomplished recently, principally by W. Maier, I. Ruf and colleagues in the examination of particular features (e.g., the chorda tympani, entotympanics) across groups of placental mammals, while discovering diagnostic features for several clades [10, 195]. Indeed, variation in chondrocranial structures is a potential source of characters for phylogenetic analyses [89, 90]. This has been barely explored in marsupials, with a few features restricted to the vomeronasal region [48, 102]. In fact, chondrocranial structures are indeed of almost no relevance in vertebrate cladistics (but see Haas [196] for a notable example). This is not surprising, as the intellectual traditions that produce these studies are quite different. The large number of discrete characters, as well as disparity in shape [197] could make the chondrocranium a source of characters for phylogenetic analyses of marsupials, but more studies and an assessment of intraspecific variation are needed. Studies of turtles, even if limited in the taxonomic scope examined, have proved to be original and worthwhile in this regard [198, 199]. Likewise, chondrocranial features and proportions have been used in discussions on the systematic allocation of vertebrate taxa (e.g., [200]).

A utilitarian argument for the study of the chondrocranium from the point of view of systematics is weak, as new diagnostic features are not likely to solve phylogenetic controversies and even the most consistent and unique of characters—should it exist, although the evidence speaks against that expectation—would be a modest part of a large data matrix of molecular data and of more easily obtained osteological and soft-tissue (e.g., myological) data. Guillerme and Cooper [201] have shown how a great number of mammalian groups are unstudied at the morphological level, and that this represents a hindrance to the incorporation of palaeontological data in systematic studies [202]. The knowledge of the chondrocranium is restricted to extant forms, as this is not preserved in fossils, however crucial to understand homologies. In summary, the study of the chondrocranium can be driven by an interest in the evolution of form and function. This needs as much or as little justification as any other intellectual pursuit.

Several authors ([1, 42, 57, 71]:165) have pointed out how some aspects of the anatomy of the chondrocranium could have functional significance for the embryo in the early postnatal life of marsupials. The area which has attracted the most attention in terms of functional morphology is that concerned with the origin of the middle ear and the transformation in the masticatory apparatus [203]. The quantification of changes in the structures involved and their spatial relations is a first step to address this subject [141], which at this point requires biomechanical studies. In this regard, how the shape of the anterior chondrocranium differs between marsupials and placentals, and among mammals of different levels of altriciality and precocity, as discussed in this paper, is also a potential subject of investigation.

Any consideration of cranial biomechanics would have to consider the architecture of muscles [204]. Much of the differentiation of craniofacial muscles in marsupials is perinatal if not postnatal, especially so in the highly altricial dasyurids, in which the “jaw muscles at birth consist of little more tan a few myotubes” ([39], p. 1186). As documented in previous works and discussed by K.K. Smith [39], differences in the timing of maturation of branchial arch muscles and the suppresion of one generation of the dentition [205] in marsupials, which evolved convergently in several placental clades [206, 207], are also fundamental aspects to consider in seeking to understand the functional morphology of early postnatal life.
